# Fast online and index-based algorithms for approximate search of RNA sequence-structure patterns

**DOI:** 10.1186/1471-2105-14-226

**Published:** 2013-07-17

**Authors:** Fernando Meyer, Stefan Kurtz, Michael Beckstette

**Affiliations:** 1 Center for Bioinformatics, University of Hamburg, Bundesstrasse 43, Hamburg 20146, Germany

## Abstract

**Background:**

It is well known that the search for homologous RNAs is more effective if both sequence and structure information is incorporated into the search. However, current tools for searching with RNA sequence-structure patterns cannot fully handle mutations occurring on both these levels or are simply not fast enough for searching large sequence databases because of the high computational costs of the underlying sequence-structure alignment problem.

**Results:**

We present new fast index-based and online algorithms for approximate matching of RNA sequence-structure patterns supporting a full set of edit operations on single bases and base pairs. Our methods efficiently compute semi-global alignments of structural RNA patterns and substrings of the target sequence whose costs satisfy a user-defined sequence-structure edit distance threshold. For this purpose, we introduce a new computing scheme to optimally reuse the entries of the required dynamic programming matrices for all substrings and combine it with a technique for avoiding the alignment computation of non-matching substrings. Our new index-based methods exploit suffix arrays preprocessed from the target database and achieve running times that are sublinear in the size of the searched sequences. To support the description of RNA molecules that fold into complex secondary structures with multiple ordered sequence-structure patterns, we use fast algorithms for the local or global chaining of approximate sequence-structure pattern matches. The chaining step removes spurious matches from the set of intermediate results, in particular of patterns with little specificity. In benchmark experiments on the Rfam database, our improved online algorithm is faster than the best previous method by up to factor 45. Our best new index-based algorithm achieves a speedup of factor 560.

**Conclusions:**

The presented methods achieve considerable speedups compared to the best previous method. This, together with the expected sublinear running time of the presented index-based algorithms, allows for the first time approximate matching of RNA sequence-structure patterns in large sequence databases. Beyond the algorithmic contributions, we provide with *RaligNAtor* a robust and well documented open-source software package implementing the algorithms presented in this manuscript. The *RaligNAtor* software is available at http://www.zbh.uni-hamburg.de/ralignator.

## Background

Due to their participation in several important molecular-biological processes, ranging from passive carriers of genetic information (tRNAs) over regulatory functions (microRNAs) to protein-like catalytic activities (Riboswitsches), non-coding RNAs (ncRNAs) are of central research interest in molceular biology [1]. NcRNAs, although synthesized as single-stranded molecules, present surprising complexity by being able to base pair with themselves and fold into numerous different structures. It is to a large extent the structure that allows them to interact with other molecules and hence to carry out various biological functions. This can also be observed in families of functionally related ncRNAs like the ones compiled in the Rfam database [2]. Here members of a family often share only few sequence features, but share by far more specific structural and functional properties. Consequently, methods for effective RNA homology search (i.e. finding new members of an RNA family) cannot rely on sequence similarity alone, but also have to take structural similarity into account.

In this paper, we address the problem of searching nucleotide databases for occurrences of RNA family members. Since for this task it is not sufficient to rely on pure sequence alignment, we briefly review search methods that employ sequence and structure information.

There exist various general sequence-structure alignment tools which determine structural similarities that are too diverse to be alignable at the sequence level. Such tools can roughly be divided into two classes. The first class consists of tools that align RNAs with given structures or determine a common structure during the alignment process. Tools like *MARNA*[3] and *RNAforester*[4] require an *a priori* known secondary structure for both input RNAs. However, they suffer from the low quality of secondary structure prediction. Addressing this problem, other tools implement variations of the Sankoff algorithm [5], which provides a general but computationally demanding solution to the problem of simultaneously computing an alignment and the common secondary structure of the two aligned sequences. Unfortunately, even tools with improved running times using variations of this algorithm (*LocARNA*[6], *Foldalign*[7,8], *Dynalign*[9,10]) or heuristics [11] are simply not fast enough for rapid searches in large nucleotide databases. Hence, in a second class we identify more specialized tools for searching RNA families in nucleotide databases. These tools use a model or motif descriptors (i.e. patterns) defining consensus sequence and secondary structure properties of the families to be searched for. For example, *Infernal*[12] and *RSEARCH*[13] infer a covariance model from a given multiple sequence alignment annotated with structure information. This model can then be used to search sequence databases for new family members. Another tool, *ERPIN*[14] is also based on automatically generated statistical secondary profiles. Although being very sensitive in RNA homology search, in particular *Infernal* and *RSEARCH* suffer from high computational demands. An alternative are tools like *RNAMotif*[15], *RNAMOT*[16], *RNABOB*[17], *RNAMST*[18], *PatScan*[19], *PatSearch*[20], or *Palingol*[21]. These methods use user-defined motif descriptors created from *a priori* knowledge about the secondary structure of the described RNA family. Another tool, *Locomotif*[22], generates a thermodynamic matcher program from a pattern drawn interactively by the user via a graphical interface. Although these tools based on motif descriptors are faster than the previously mentioned tools, they have a running time that scales at least linearly with the size of the target sequence database. This makes their application to large databases challenging. Previously, we addressed this problem by presenting *Structator*[23], an ultra fast index-based bidirectional matching tool that achieves sublinear running time by exploiting base pair complementarity constraints for search space reduction.

Apart from running time constraints, another major disadvantage of all current tools that search for sequence-structure patterns is their limited capacity to find approximate matches to the patterns. Although variability in length of pattern elements is often allowed, this is constrained to certain pattern positions that must be specified by the user. This limitation also holds for our *Structator* tool. Also, variations (insertions, deletions, or replacements) in the sequence that lead to small structural changes, such as the breaking of a base pair, are not supported. This often hampers the creation of patterns that are specific but generalized enough to match all family members. An algorithm presented in [24] only partially alleviates this problem by finding approximate matches of a helix in a genome allowing edit operations on single bases, but not on the structure.

To overcome these issues, we present new fast index-based and online algorithms for approximate matching of sequence-structure patterns, all implemented in an easy-to-use software package. Given one or more patterns describing any (branching, non-crossing) RNA secondary structure, our algorithms compute alignments of the complete patterns to substrings of the target sequence, i.e. semi-global alignments, taking sequence and structure into account. For this, they apply a full set of edit operations on single bases and base pairs. Matches are reported for alignments whose sequence-structure edit cost and number of insertions and deletions do not exceed user-defined thresholds. Our most basic algorithm is a scanning variant of the dynamic programming algorithm for global pairwise sequence-structure alignment of Jiang *et al.*[25], for which no implementation was available. Because its running time is too large for database searches on a large scale, we present accelerated online and index-based algorithms. All our new algorithms profit from a new computing scheme to optimally reuse the required dynamic programming matrices and a technique to save computation time by determining as early as possible whether a substring of the target sequence can contain a match. In addition, our index-based algorithms employ the suffix array data structure compiled from the search space. This further reduces the running time.

As in [23], we also support the description of an RNA molecule by multiple ordered sequence-structure patterns. In this way, the molecule’s secondary structure is decomposed into a sequence of substructures described by independent sequence-structure patterns. These patterns are efficiently aligned to the target sequences using one of our new algorithms and the results are combined with fast global and local chaining algorithms [23,26]. This allows a better balancing of running time, sensitivity, and specificity compared to searching with a single long pattern describing the complete sequence and secondary structure.

Before we describe our algorithms, we formalize the approximate search problem with the involved sequence-structure edit operations. Then we present, step by step, two efficient online and two index-based matching algorithms. We proceed with a short review of the approach for computing chains of matches. Finally, we present several benchmark experiments.

## Methods

### Preliminaries

An RNA *sequenceS* of length *n* = |*S*| over the set of bases A={A, C, G, U} is a juxtaposition of *n* bases from A. *S*[*i*], 1 ≤ *i* ≤ *n*, *denotes the* *base of* *S at position* *i*. Let *ε* denote the empty sequence, the only sequence of length 0. By $A^{n}$ we denote the set of sequences of length *n* ≥ 0 over A. The set of all possible sequences over A including the empty sequence *ε* is denoted by $A^{*}$.

For a sequence *S* = *S*[1] *S*[2] … *S*[*n*] and 1 ≤ *i* ≤ *j* ≤ *n*, *S*[*i*..*j*] denotes the *substring* *S*[*i*] *S*[*i* + 1] … *S*[*j*] of *S*. For *S* = *uv*, *u* and $v\in A^{*}$, *u* is a *prefix* of *S*, and *v* is a *suffix* of *S*. The *k*–th suffix of *S* starts at position *k*, while the *k*–th prefix of *S* ends at *k*. For 1 ≤ *k* ≤ *n*, *S*_*k*_ denotes the *k*–th suffix of *S*. For stating the space requirements of our index structures, we assume that |*S*| < 2^32^, so that sequence positions and lengths can be stored in 4 bytes.

The secondary structure of an RNA molecule is formed by Watson-Crick pairing of complementary bases and also by the slightly weaker wobble pairs. We say that two bases (c,d)∈A×A are *complementary* and can form a *base pair* if and only if (c,d)∈C={(A, U), (U, A), (C, G), (G, C), (G, U), (U, G)}. If two bases *a* and *b* form a base pair we also say that there exists an *arc* between *a* and *b*. A *non-crossing RNA structure*$\hat{R}$*of length* *m* is a set of *base pairs* (*i*,*j*), 1 ≤ *i* < *j* ≤ *m*, stating that the base at position *i* pairs with the base at position *j*, such that for all $(i,j),(i^{\prime },j^{\prime })\in \hat{R}:i<i^{\prime }<j^{\prime }<j$ or *i*^′^ < *i* < *j* < *j*^′^ or *i* < *j* < *i*^′^ < *j*^′^ or *i*^′^ < *j*^′^ < *i* < *j*. A standard notation for $\hat{R}$ is a *structure string R* over the alphabet {**.,(,)**} such that for each base pair $(i,j)\in \hat{R}$, *R*[*i*] = ***(*** and *R*[*j*] = ***)***, and *R*[*r*] = ***.*** for positions *r*, 1 ≤ *r* ≤ *m*, that do not occur in any base pair of $\hat{R}$, i.e. *r* ≠ *i* and *r* ≠ *j* for all $(i,j)\in \hat{R}$.

Let Φ = {R, Y, M, K, W, S, B, D, H, V, N} be a set of characters. According to the IUPAC definition, each character in Φ denotes a specific character class φ(x)⊆A. Each character x∈A can be seen as a character class *φ*(*x*) = {*x*} of exactly one element. A *sequence pattern* is a sequence $P\in {\left(A\cup \Phi \right)}^{*}$. An *RNA sequence-structure pattern (RSSP)*Q=(P,R) of length *m* is a pair of a *sequence pattern* *P* and a *structure string* *R*, both of length *m*. With Q[i..j] we denote the RSSP region (*P*[*i*..*j*],*R*[*i*..*j*]).

### Approximate matching of RNA sequence-structure patterns

To find in a long RNA sequence *S* approximate matches of an RSSP Q describing a part of an RNA molecule, we compute alignments of the complete Q and substrings of *S* considering edit operations for unpaired bases and base pairs. That is, we compute semi-global alignments simultaneously obtaining the sequence-structure edit distance of Q and substrings of *S*.

**Figure 1:**
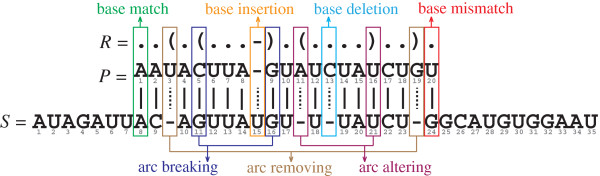
**Example of a semi-global alignment of a sequence-structure pattern**Q=(P,R)** and an RNA sequence *S* and involved sequence-structure edit operations.** Continuous (dashed) lines indicate match (gap) alignment edges from *A*_match_ (*A*_gap_).

We define the alignment of Q and a substring *S*[*p*..*q*], 1 ≤ *p* ≤ *q* ≤ *n*, as set *A* = *A*_match_ ⊎ *A*_gap_. The set *A*_match_ ⊆ [1..*m*] × [*p*..*q*] of match edges satisfies that, for all different (*k*,*l*),(*k*^′^,*l*^′^) ∈ *A*_match_, *k* > *k*^′^ implies *l* > *l*^′^. The set *A*_gap_ of gap edges is defined as $\left\{(x,-)|x\in [1\text{..m}]\wedge ∄y,(x,y)\in A_{\text{match}}\}\cup \{(-,y)|y\in [\text{p..q}]\wedge ∄x,(x,y)\in A_{\text{match}}\right\}$. See Figure 1 for an example of a semi-global alignment and associated alignment edges. The alignment cost is based on a sequence-structure edit distance. The allowed edit operations on unpaired bases *P*[*k*] and *S*[*l*], 1 ≤ *k* ≤ *m*, *p* ≤ *l* ≤ *q*, are *base mismatch* (*match*), with cost *ω*_m_ (zero), which occurs if there is an edge (*k*,*l*) ∈ *A*_match_ and *S*[*l*] ∉ *φ*(*P*[*k*]) (*S*[*l*] ∈ *φ*(*P*[*k*])), and *base deletion* (*insertion*), with cost *ω*_d_, which occurs if (*k*,−) ∈ *A*_gap_ ((−,*l*) ∈ *A*_gap_). The possible edit operations on base pairs were first introduced by Jiang *et al.*[25] and are defined as follows. Let (*k*_1_,*k*_2_) be a base pair in $\hat{R}$ and *l*_1_ and *l*_2_, *p* ≤ *l*_1_ < *l*_2_ ≤ *q*, be positions in *S*. 

• An *arc breaking*, with cost *ω*_b_, occurs if (*k*_1_,*l*_1_) ∈ *A*_match_ and (*k*_2_,*l*_2_) ∈ *A*_match_ but bases *S*[*l*_1_] and *S*[*l*_2_] are not complementary. An additional base mismatch cost *ω*_m_ is caused if *S*[*l*_1_] ∉ *φ*(*P*[*k*_1_]) and another if *S*[*l*_2_] ∉ *φ*(*P*[*k*_2_]). To give an example, consider the semi-global alignment in Figure 1. RSSP Q contains base pair $(5,9)\in \hat{R}$ and there exist edges (5,11) ∈ *A*_match_ and (9,16) ∈ *A*_match_ but *S*[11] = *G* and *S*[16] = *G* are not complementary. We note a difference between our definition and the definition of Jiang *et al.*, where both aligned sequences are annotated with structure information. There, an arc breaking occurs if bases *S*[*l*_1_] and *S*[*l*_2_] are annotated as unpaired in addition to the condition of existing edges (*k*_1_,*l*_1_) ∈ *A*_match_ and (*k*_2_,*l*_2_) ∈ *A*_match_. Hence, because in our case sequence *S* has no structure annotation, our definition is based on the complementarity of bases *S*[*l*_1_] and *S*[*l*_2_].

• An *arc altering*, with cost *ω*_a_, occurs if either (1) (*k*_1_,*l*_1_) ∈ *A*_match_ and (*k*_2_,−) ∈ *A*_gap_ or (2) (*k*_2_,*l*_2_) ∈ *A*_match_ and (*k*_1_,−) ∈ *A*_gap_. Each case induces an additional base mismatch cost *ω*_m_ if *S*[*l*_1_] ∉ *φ*(*P*[*k*_1_]) or *S*[*l*_2_] ∉ *φ*(*P*[*k*_2_]). As an example, observe in the alignment shown in Figure 1 that there exist a base pair $(11,16)\in \hat{R}$ and edges (11,−) ∈ *A*_gap_ and (16,21) ∈ *A*_match_.

• An *arc removing*, with cost *ω*_r_, occurs if (*k*_1_,−) ∈ *A*_gap_ and (*k*_2_,−) ∈ *A*_gap_. As an example, observe in the alignment in Figure 1 that there exist a base pair $(3,19)\in \hat{R}$and edges (3,−) ∈ *A*_gap_ and (19,−) ∈ *A*_gap_.

With this set of edit operations on the sequence and structure we can now define the cost of the alignment of Q and *S*[*p*..*q*] as

(1) $$\begin{array}{l} \text{dist}(Q,S[\text{p..q}])=\text{min}\{\text{dis}t_{A}(Q,S[\text{p..q}])\;|\;A\\ \text{is an alignment of}\;Q\;\text{and}\;S[\text{p..q}]\} \end{array}$$

where

(2) $$\begin{array}{l} \begin{array}{lcl} & \text{dis}t_{A}(Q,S[\text{p..q}])= & \\ & \underset{\left(k,l)\in A,R[k]=.,S[l]\notin \varphi (P[k]\right)}{\sum }\omega_{m} & \text{base mismatch}\\ + & \underset{(k,-)\in A,R[k]=.}{\sum }\omega_{d} & \text{base deletion} \end{array}\\ \begin{array}{lcl} + & \underset{(-,l)\in A}{\sum }\omega_{d} & \text{base insertion}\\ + & \underset{(k_{1},k_{2})\in \hat{R},(k_{1},l_{1})\in A,(k_{2},l_{2})\in A,(S[l_{1}],S[l_{2}])\notin C}{\sum }\omega_{b} & \text{arc breaking}\\ + & \underset{(k_{1},k_{2})\in \hat{R},(k_{1},l_{1})\in A,(k_{2},-)\in A}{\sum }\omega_{a} & \text{arc altering}\\ + & \underset{(k_{1},k_{2})\in \hat{R},(k_{2},l_{2})\in A,(k_{1},-)\in A}{\sum }\omega_{a} & \text{arc altering}\\ + & \underset{(k_{1},k_{2})\in \hat{R},(k_{1},-)\in A,(k_{2},-)\in A}{\sum }\omega_{r} & \text{arc removing}. \end{array} \end{array}$$

An alignment *A* of minimum cost between Q and *S*[*p*..*q*] is an *optimal alignment* of Q and *S*[*p*..*q*].

In practice, one is often interested in finding substrings of an RNA sequence *S* having a certain degree of similarity to a given RSSP Q on both the sequence and structure levels. Therefore, we are only concerned about optimal alignments of Q and substrings *S*[*p*..*q*] with up to a user-defined sequence-structure edit distance and a limited number of allowed insertions and deletions (indels). More precisely: 

• the cost dist(Q,S[p..q]) should not exceed a given threshold K, and

• the number of indels in the alignment should be at most *d*.

Thus, the approximate search problem for finding occurrences of an RSSP Q in *S*, given user-defined thresholds K and *d*, is to report all intervals [*p*..*q*] such that

(3) dist(Q,S[p..q])≤Kandm−d≤|S[p..q]|≤m+d≤n.

We call every substring *S*[*p*..*q*] satisfying Equation (3) a *match* of Q in *S*. In the subsequent sections we present algorithms for searching for matches of an RSSP Q in a sequence *S*.

### Online approximate RNA database search for RSSPs: *ScanAlign*

A straightforward algorithm to search for approximate matches of an RSSP Q in an RNA sequence *S* consists of sliding a window of length *m*^′^ = *m* + *d* along *S* while computing dist(Q,S[p..q]) for 1 ≤ *p* ≤ *q* ≤ *n* and *q* − *p* + 1 = *m*^′^. We note that, although the length of a match can vary in the range *m* − *d* to *m* + *d*, to find matches of all possible lengths it suffices to slide a window of length *m*^′^ along *S* corresponding to substrings *S*[*p*..*q*]. This holds because the alignment to a window of length *m*^′^ entails all possible alignments with up to *d* allowed indels. In the following we present a dynamic programming algorithm computing dist(Q,S[p..q]) for every window *S*[*p*..*q*]. Our recurrences are derived from the algorithm for global pairwise sequence-structure alignment of Jiang *et al.*[25], i.e. an algorithm for aligning sequences of similar lengths. Although Jiang’s algorithm supports the sequence-structure edit operations described above, we emphasize that it is not suitable for computing semi-global alignments, which is what we are interested in.

We begin the description of our algorithm by defining three functions required by the dynamic programming recurrences. Let *T* = *S*[*p*..*q*]. 

1. For computing base match and mismatch costs for positions *i* and *j* of the RSSP Q=(P,R) and substring *T*, respectively, we define a function χ:N×N→{0,1} as:

(4) $$\begin{array}{llll} \chi (i,j)=\left\{\begin{array}{lll} 0 & \text{if}\;T[j]\in \varphi (P[i]) & \text{(base match)}\\ 1 & \text{otherwise.} & \text{(base mismatch)} \end{array}\right) & & & \end{array}$$

2. To determine whether an arc breaking operation can occur, we must also be able to check for base complementarity at positions *i* and *j* of *T*. Therefore, we define a function comp:N×N→{0,1} as:

(5) $$\begin{array}{l} \text{comp}(i,j)=\left\{\begin{array}{lll} 0 & \text{if}\;(T[i],T[j])\in C & \text{(complementary)}\\ 1 & \text{otherwise.} & \text{(not complementary)} \end{array}\right) \end{array}$$

3. For determining the correct row (of the dynamic programming matrices introduced below) where certain operation costs must be stored we introduce a function row:N→N defined as:

(6) $$\text{row}(i)=\left\{\begin{array}{ll} i^{\prime } & \text{if}(i^{\prime },i)\in \hat{R}\;\text{and}\;1<i^{\prime }<i<m\;\text{and}\;R[i+1]\\ & =.\;\text{and}\;R[i^{\prime }-1]\neq \text{(}\\ 0 & \text{if}\;(i,i^{\prime })\in \hat{R}\;\text{and}\;R[i+1]=.\\ i & \text{otherwise}. \end{array}\right)$$

Intuitively, function *row* satisfies the following: (1) given the right index *i* of a base pair (*i*^′^,*i*), it returns the left index *i*^′^ if (*i*^′^,*i*) is preceded or followed by other structures; (2) given the left index *i* of a base pair (*i*,*i*^′^), it returns 0 if the base at position *i* + 1 of Q is unpaired; and (3) given any other position index *i*, it returns *i* itself.

Using these three functions, our algorithm determines the sequence-structure edit distance $\text{dist}(Q,T[1\mathrm{..}m^{\prime }])$ by computing a series of *m*^′^ + 1(*m*^′^ + 1) × (*m*^′^−*k* + 1) matrices *DP*_*k*_, for 1 ≤ *k* ≤ *m*^′^ + 1, such that $DP_{1}(\text{row}(m),m^{\prime })=\text{dist}(Q,T[1\mathrm{..}m^{\prime }])$. We remark that *DP*_*k*_(*i*,*j*) is not defined for every subinterval [*i*..*j*]. While the recurrences of Jiang’s algorithm are divided in four main cases, we present a simplified recurrence relation with only two main cases. In addition, we observe that we use only three indices for a matrix entry instead of four. Our recurrences are as follows. 

1. If *i* = 0 or *R*[*i*] =. (unpaired base), then

(7) $$\begin{array}{l} \begin{array}{l} DP_{k}(i,j)=\left\{\begin{array}{ll} 0 & \text{if}\;i=0\;\text{and}\;j=0\\ DP_{k}(0,j-1)+\omega_{d} & \text{if}\;i=0\;\text{and}\;j>0\\ DP_{k}(\text{row}(i-1),0)+\omega_{d} & \text{if}\;i>0\;\text{and}\;j=0\\ \text{min}\left\{\;\begin{array}{l} DP_{k}(\text{row}(i-1),j)+\omega_{d}\\ DP_{k}(i,j-1)+\omega_{d}\\ DP_{k}(\text{row}(i-1),j-1)+\chi (i,j)\omega_{m} \end{array}\right\} & \text{if}\;i>0\;\text{and}\;j>0 \end{array}\right) \end{array} \end{array}$$

2. If *R*[*i*] ≠. (paired base), then

(a) If *R*[*i*] =) where *i* forms base pair $(i^{\prime },i)\in \hat{R}$,

(8) $$\begin{array}{l} DP_{k}(i,j)=\left\{\begin{array}{ll} DP_{k}(\text{row}(i-1),0)+\omega_{r} & \text{if}\;j=0\\ \text{min}\left\{\begin{array}{l} DP_{k}(\text{row}(i-1),j-1)+\chi (i,j+k)\omega_{m}+\omega_{a}\\ DP_{k+1}(\text{row}(i-1),j-1)+\chi (i^{\prime },k)\omega_{m}+\omega_{a}\\ DP_{k}(\text{row}(i-1),j)+\omega_{r}\\ DP_{k}(i,j-1)+\omega_{d}\\ DP_{k+1}(i,j-1)+\omega_{d}\\ DP_{k+1}(\text{row}(i-1),j-2)+(\chi (i,j+k)\\ \;+\chi (i^{\prime },k+1))\omega_{m}+\\ \text{comp}(k+1,j+k)\omega_{b},\;\text{if}\;j>1 \end{array}\right\} & \text{if}\;j>0 \end{array}\right) \end{array}$$

(b) If (a) holds and either *R*[*i*^′^−1] =. or *R*[*i*^′^−1] =), compute in addition to Equation (8)

(9) $$DP_{k}(\text{row}(i),j)=\left\{\begin{array}{ll} DP_{k}(\text{row}(i^{\prime }-1),0)+DP_{k}(i,0) & \text{if}\;j=0\\ \text{min}\left\{DP_{k}(\text{row}(i^{\prime }-1),j^{\prime })+DP_{k+j^{\prime }}(i,j-j^{\prime })|0\leq j^{\prime }\leq j\right\} & \text{if}\;j>0 \end{array}\right)$$

A natural way to compute these *DP* matrices is top down, checking whether case 1, 2(a), or 2(b) applies, in this order. Due to the matrix dependencies in cases 2(a) and (b), the matrices need to be computed simultaneously.

Note that for all *j*, 1 ≤ *j* ≤ *m*^′^, clearly $DP_{1}(\text{row}(m),j)=\text{dist}(Q,T[1\text{..j}])$. Therefore all candidate matches shorter than *m*^′^ beginning at position *p* are also computed in the computation of $\text{dist}(Q,T[1\mathrm{..}m^{\prime }])$. The following Lemma is another important contribution of this work and also the key for the development of an efficient algorithm.

#### Lemma 1

*When sliding a window along S to compute*dist(Q,S[p..q]), 1 ≤ *p* ≤ *q* ≤ *n*, *m*^′^ = *q* − *p* + 1 = *m* + *d*, *a window shift by one position to the right requires to compute only column m*^′^−*k* + 1, *i.e. the last column of matrices DP*_*k*_, 1 ≤ *k* ≤ *m*^′^.

#### Proof

Let *T*[1..*m*^′^] = *S*[*p*..*q*]. The computation of $\text{dist}(Q,T[1\mathrm{..}m^{\prime }])$ requires to compute *m*^′^ + 1 *DP* matrices, one for each suffix *T*_*k*_ of string *T* = *T*[1..*m*^′^], 1 ≤ *k* ≤ *m*^′^, and one for the empty sequence *ε*. As a result, it holds for every *k* that $\text{dist}(Q,T_{k})=DP_{k}(\text{row}(m),m^{\prime })$ which is obtained as a by-product of the dist(Q,T) computation. Because each substring *T*_*l*+1_[1..*m*^′^−*l*] = *S*[*p* + *l*..*q*], 0 ≤ *l* < *m*^′^, only differs by its last character from *S*[*p* + *l* + 1..*q* + 1] which are suffixes of the window substring shifted by one position to the right, the lemma holds. □

**Figure 2:**
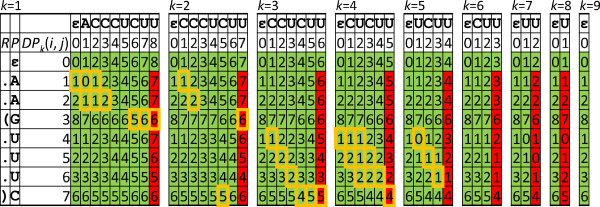
***DP* tables for the sequence-structure alignment computation of RSSP**Q=(AAGUUUC,..(...))** and window substring *T *= ACCCUCUU when scanning a sequence *S * with algorithm *ScanAlign *.** Only the entries in red have to be computed for each window shift, whereas the entries in green are reused. Entries in yellow boxes are on a possible minimizing path for alignments with up to *d* = 1 indels. The following operation costs were used: *ω*_d_ = *ω*_m_ = 1, *ω*_b_ = *ω*_a_ = 2, and *ω*_r_ = 3.

Due to Lemma 1, our algorithm computes only the last column of the *DP* matrices for every shifted window substring (see the example in Figure 2) and just for the first window *S*[1..*m*^′^] it computes every column. We call this algorithm *ScanAlign*. We note that during the reviewing process of this manuscript, Will *et al.*[27] submitted and published an algorithm for semi-global sequence-structure alignment of RNAs. As our method, this algorithm saves computation time by reusing entries of dynamic programming tables while scanning the target sequence.

Our *ScanAlign* algorithm has the following time complexity: computing *DP*_*k*_(*i*,*j*) in cases 1 and 2(a) takes *O*(1) time and in case 2(b) it takes *O*(*m*^′^) time. Now consider the two situations: 

• For the first computed window substring *S*[1..*m*^′^], cases 1 and 2(a) require *O*(*mm*^′2^) time in total and case 2(b) requires *O*(*mm*^′3^) time in total. This leads to an overall time of *O*(*mm*^′3^).

• For one window shift, cases 1 and 2(a) require *O*(*mm*^′^) time in total and case 2(b) requires *O*(*mm*^′2^) time in total, leading to an overall time of *O*(*mm*^′2^).

Since there are *n* − *m*^′^−1 window shifts, the computation for all shifted windows takes *O*(*mm*^′2^(*n*−*m*^′^)) = *O*(*mm*^′2^*n*) time. We observe that the time needed by *ScanAlign* to compute all window shifts reduces to *O*(*mm*^′^*n*) if recurrence case 2(b) is not required. This is the case if the structure of Q does not contain unpaired bases before a base pair constituting e.g. a left dangling end or left bulge.

### Faster online alignment with early-stop computation: LScanAlign

Often, before completing the computation of the alignment between an RSSP Q and a window substring *S*[*p*..*q*] of the searched RNA sequence, we can determine whether the cost of this alignment will exceed the cost threshold K. By identifying this situation as early as possible, we can improve algorithm *ScanAlign* to skip the window, thus saving computation time and proceed with aligning the next window. The idea consists in checking, during the alignment computation, whether the cost of an already aligned region of Q and a substring of *S*[*p*..*q*] exceeds K. In such a case, the alignment cost of the complete Q and *S*[*p*..*q*] will also exceed K. In more detail, this works as follows. 

• We decompose the RSSP Q into regions that can themselves represent a pattern, e.g. a stem-loop or unpaired region. A basic constraint is to not split base pairs to different regions.

• We compute the alignment of a given initial RSSP region and a substring of the current window *S*[*p*..*q*], progressively extending the alignment to other regions.

• If the cost of aligning an RSSP region to a substring of the window exceeds cost threshold K, then the entire pattern cannot match the window. This means that the window can immediately be skipped.

Formally, a valid RSSP region Q[x..y], 1 ≤ *x* ≤ *y* ≤ *m*, satisfies exactly one of the following conditions. 

**Figure 3:**
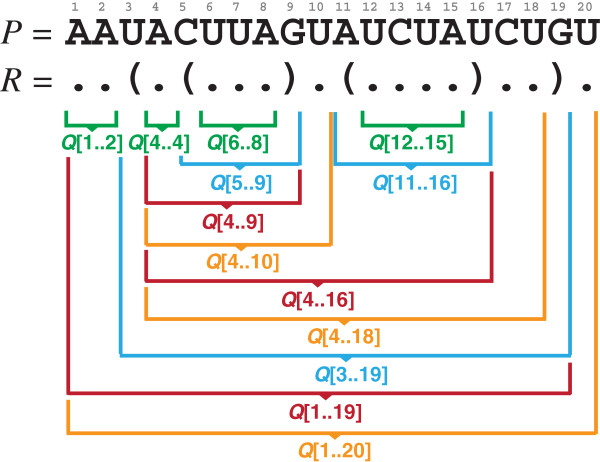
**Regions of RSSP**Q=(AAUACUUAGUAUCUAUCUGU,..(.(...).(....)..).)** according to conditions 1 (green), 2 (orange), 3 (blue), and 4 (red) described in the text.**

1. Q[x..y] is a left dangling (unpaired) end of the pattern in 5^′^ to 3^′^ direction, i.e. *x* = 1. Alternatively, it is an unpaired region of maximal length such that position *x* − 1 forms a base pair $(x-1,y^{\prime })\in \hat{R}$ for some position *y*^′^ of Q. Observe that no extension of Q[x..y] by another unpaired position is possible. As an example, consider the green marked regions Q[1..2], Q[4..4], Q[6..8], and Q[12..15] in Figure 3.

2. Position *y* is unpaired and there is at least one base pair $(x^{\prime },y^{\prime })\in \hat{R}$, *x* ≤ *x*^′^ < *y*^′^ < *y*. No extension of Q[x..y] by another unpaired position is possible. As examples of regions under these requirements, see the regions in orange of the RSSP Q in Figure 3, namely Q[4..10], Q[4..18], and Q[1..20].

3. $(x,y)\in \hat{R}$ is a base pair. For examples of such RSSP regions, see the regions in blue of the RSSP in Figure 3, namely Q[5..9], Q[11..16], and Q[3..19].

4. *y* forms a base pair $(x^{\prime },y)\in \hat{R}$ where either *R*[*x*^′^−1] =. or *R*[*x*^′^−1] =), 1 ≤ *x* ≤ *x*^′^−1. In addition, *x* = 1 or $(x-1,y^{\prime })\in \hat{R}$ for some *y*^′^ > *y*. Examples of such RSSP regions are shown in red in Figure 3, i.e. regions Q[4..9], Q[4..16], and Q[1..19].

Note that regions can be embedded in other regions but cannot partially overlap another.

Our progressive alignment computation of an RSSP Q and a window substring of the searched RNA sequence *S* begins by considering only an in general small region of Q embedded in another region. The computation is then extended to a surrounding region, e.g. from region Q[6..8] to Q[5..9] of the RSSP shown in Figure 3, until it entails the largest region surrounding all other regions, e.g. Q[1..20] of the same example. Formally, we elaborate the alignment computation as follows. Let *T* = *T*[1..*m*^′^] be a window substring of length *m*^′^ = *m* + *d* of *S* and *d* be the number of allowed indels. Pattern regions have the property that, for any region Q[x..y], computing dist(Q[x..y],T) does not depend on any other region $Q[x^{\prime }\mathrm{..}y^{\prime }]$ for some *y*^′^ < *x* and *x*^′^ < *y*. Therefore, they can easily be sorted to indicate the order by which the rows of the *DP* matrices are computed. We observe that the top-down computation of the *DP* matrices, as described above, automatically sorts the regions and respects the dependency between rows. To obtain from the sorted regions the indices of the rows to be computed, we consider the condition satisfied by each region. The rows obtained according to each condition are computed according to one case of the recurrence. Given region Q[x..y] identified by one of the four conditions this region satisfies, the following rows of the matrices have to be computed. 

1. All rows in the interval [*x*..*y*] are computed by Equation (7).

2. One scans the structure of region Q[x..y] from position *y* to position *x* until one finds a paired position *y*^′^. Then, all rows in the interval [*y*^′^ + 1..*y*] are computed by Equation (7).

3. Row *y* is computed by recurrence (a) of Equation (8).

4. Row *row*(*y*) is computed by recurrence (b) of Equation (8).

The sequential computation of the rows belonging to each region naturally leads to the computation of the entire alignment of Q and sequence-structure edit distance dist(Q,T).

Our improvement of the *ScanAlign* algorithm is based on the following two observations. 

• The standard dynamic programming algorithm for aligning two plain text sequences of lengths *m* and *n* requires an (*m* + 1) × (*n* + 1) matrix. Let *i* and *j* be indices of each of the matrix dimensions and a diagonal *v* be those entries defined by *i* and *j* such that *j* − *i* = *v*. Given that the cost of each edit operation is a positive value, the cost of the entries along a diagonal of the matrix are always non-decreasing [28].

• Moreover, one indel operation implies that an optimal alignment path including an entry on diagonal *v* also includes at least one entry on diagonal *v* + 1 or *v* − 1. Now let *v* be the diagonal ending at the entry on the lower-right corner of the matrix and *d* be the number of allowed indels. One can stop the alignment computation as soon as all the entries of one row in the matrix and along diagonals *v* + *d*^′^, −*d* ≤ *d*^′^ ≤ *d*, exceed K.

For our improvement of algorithm *ScanAlign*, based on the following Lemma, we define a diagonal for each RSSP region instead of only one for the entire matrices.

#### Lemma 2

*Assume an RSSP*Q=(P,R), *a region*Q[x..y]*of length l* = *y* − *x* + 1, *a window substring T*[1..*m*^′^] *of the searched RNA sequence, a cost threshold*K, *and number d of allowed indels. If for every d*^′^, −*d* ≤ *d*^′^ ≤ min{*d*,*x*}, *z* ∈ {|*d*^′^|−*d*,−|*d*^′^|+*d*}, *y* + *d*^′^ ≤ *m*^′^, *it holds that*$\text{dist}(Q[\text{x..y}],T_{x+d^{\prime }}[1\mathrm{..l}+z])>k$, *then, for every d*^′′^, 0 ≤ *d*^′′^ ≤ *d*, $\text{dist}(Q,T[1\mathrm{..}m^{\prime }-d^{\prime \prime }])>k$.

#### Proof

If the RSSP region Q[x..y] originates from condition 1 or 2 (3 or 4) above, we define the entries on a diagonal *e* as those entries *DP*_*k*_(*i*,*j*) (*DP*_*k*_(*row*(*y*),*j*)), 1 ≤ *k* ± *d* ≤ *m*^′^, such that *j* − *i* + *offset* = *e*, where *offset* = *x* − 1. Without loss of generality let *d* = 1. Assuming *x* − 1 > 0 and *y* + 1 ≤ *m*^′^, this means that an optimal alignment of pattern Q and substring *T* requires Q[x..y] to align with: 

• *T*[*x*..*y*], *T*[*x*..*y* − 1], or *T*[*x*..*y* + 1], requiring for all three alignments the computation of $\text{dist}(Q[\text{x..y}],T_{x}[1\mathrm{..l}+z])$ for *z* ∈ {0−1,0 + 1} = {−1,1};

• *T*[*x* − 1..*y* − 1], requiring the computation of $\text{dist}(Q[\text{x..y}],T_{x-1}[1\mathrm{..l}+z])$ for *z* ∈ {|−1|−1,−|−1| + 1} = {0}; or

• *T*[*x* + 1..*y* + 1], requiring the computation of $\text{dist}(Q[\text{x..y}],T_{x+1}[1\mathrm{..l}+z])$for *z* ∈ {|1|−1,−|1| + 1} = {0}.

The alignments with *T*[*x*..*y*], *T*[*x*..*y* + 1], and *T*[*x*..*y*−1] end in matrix *DP*_*x*_. The alignments with *T*[*x*−1..*y*−1] end in matrix *DP*_*x*−1_, and the alignments with *T*[*x* + 1..*y* + 1] end in matrix *DP*_*x*+1_. Every minimizing path obtained for the entire alignment of Q and *T* can only include the entries on the diagonals *e*, *e* + 1, and/or *e* − 1 for the alignments with *T*[*x*..*y*], *T*[*x*..*y* + 1], and *T*[*x*..*y* − 1], and can only include the entries on diagonal *e* for the alignments with *T*[*x* − 1..*y* − 1] and *T*[*x* + 1..*y* + 1] because these substrings already imply alignments with one indel. As the sum of the cost of the edit operations on the minimizing path increases monotonically and there cannot be other minimizing paths due to the limited number of indels *d*, the lemma holds. □

Let Q be an RSSP whose regions are sorted by the order of computation of their respective rows in the *DP* tables above, let *d* be the number of allowed indels, and *T* = *T*[1..*m*^′^] be a window substring of the searched RNA sequence. Applying Lemma 2, we modify algorithm *ScanAlign* to compute the alignment of each region Q[x..y] to substrings $T_{x+d^{\prime }}$, −*d* ≤ *d*^′^ ≤ min{*d*,*x*}, *y* + *d*^′^ ≤ *m*^′^, and progressively extend the alignment to other RSSP regions and substrings of *T* as long as $\text{dist}(Q[\text{x..y}],T_{x+d^{\prime }}[1\mathrm{..l}+z])\leq k$, *z* ∈ {|*d*^′^|−*d*,−|*d*^′^| + *d*}, holds. That is, for each RSSP region, it determines the rows and recurrence case required for their computation according to conditions 1, 2, 3, or 4 above. Then, within each processed row *i*, it checks whether for at least one entry *DP*_*k*_(*i*,*j*) on a possible minimizing path, i.e. on diagonals *e*^′^, *e* − *d* ≤ *e*^′^ ≤ *e* + *d*, *DP*_*k*_(*i*,*j*) ≤ *k*. If no entry is below K, it skips the alignment computation for all remaining RSSP regions and proceeds with aligning the next window. See Figure 2 for an example of the *DP* matrices of an alignment computation whose entries on a possible minimizing path are highlighted in yellow.

When scanning the searched RNA sequence, a window can be shifted before all *DP* matrices entries are computed. Hence, a direct application of Lemma 1 is no longer possible. To overcome this, we define an array *Z* in the range 1 to *z*, where *z* is the number of RSSP regions, and associate each region with an index *r*, 1 ≤ *r* ≤ *z*. Let *p* be the starting position of the window substring *S*[*p*..*q*] in the RNA sequence. We set *Z*[*r*] = *p* whenever all *DP* matrices rows and columns belonging to region *r* are computed. This occurs when the cost of aligning this region does not exceed cost threshold K. Now, when aligning the same RSSP region *r* to a different window substring *S*[*p*^′^..*q*^′^], *p*^′^ > *p*, computing all *DP* matrices columns requires to compute the last *p*^′^ − *p* columns. If *p*^′^ − *p* < *m*^′^ (recall that *m*^′^ = *q* − *p* = *q*^′^−*p*^′^), this means that the two window substrings do not overlap and therefore no *DP* matrix column can be reused.

Our improved algorithm, hereinafter called *LScanAlign*, in the worst case needs to process every RSSP region for every window shift. Hence, it has the same time complexity as algorithm *ScanAlign*. However, as in many cases only a few RSSP regions are evaluated, it is much faster in practice as will be shown later. *ScanAlign* and *LScanAlign* are the basis for further improvements presented in the subsequent sections.

### Index-based search: LESAAlign

Suffix trees and enhanced suffix arrays are powerful data structures for exact string matching and for solving other string processing problems [29,30]. In the following we show how the use of enhanced suffix arrays leads to even faster algorithms for searching for matches of an RSSP Q in an RNA sequence *S*.

**Figure 4:**
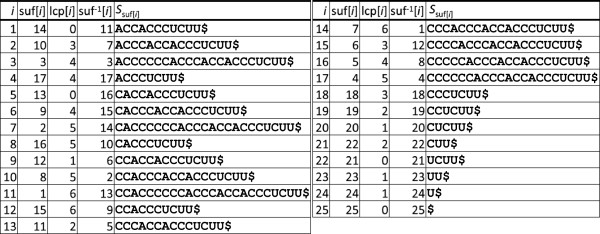
**Enhanced suffix array of sequence *S*$ = CCACCCCCCACCCACCACCCUCUU$ consisting of the suffix array ****suf****, longest common prefix array ****lcp****, and inverse suffix array ****suf**^−1^**.** For the definition of suf^−1^, see the section describing algorithm *LGSlinkAlign*.

The enhanced suffix array of a sequence *S* is composed of the suffix array suf and the longest common prefix array lcp. Let $, called terminator symbol, be a symbol not in A for marking the end of a sequence. $ is larger than all the elements in A. suf is an array of integers in the range 1 to *n* + 1 specifying the lexicographic order of the *n* + 1 suffixes of the string *S*$. That is, *S*_suf__[1]_,*S*_suf__[2]_,...,*S*_suf__[*n*+1]_ is the sequence of suffixes of *S* in ascending lexicographic order. Table suf requires 4*n* bytes and can be constructed in *O*(*n*) time and space [31]. In practice non-linear time construction algorithms [32,33] are often used as they are faster. lcp is a table in the range 1 to *n* + 1 such that lcp[1] = 0, and lcp[*i*] is the length of the longest common prefix between *S*_suf__[*i*−1]_ and *S*_suf__[*i*]_ for 1 < *i* ≤ *n* + 1. Table lcp requires *n* bytes and stores entries with value up to 255, whereas occasional larger entries are stored in an exception table using 8 bytes per entry [30]. More space efficient representations of the lcp table are possible (see [34]). The construction of table lcp can be accomplished in *O*(*n*) time and space given suf[35]. For an example of an enhanced suffix array, see Figure 4. In the following we assume that the enhanced suffix array of *S* has already been computed.

Consider an RSSP Q to be matched against an RNA sequence *S* with up to *d* indels. For each *i*, 1 ≤ *i* ≤ *n*, let *p*_*i*_ = min{*m* + *d*,|*S*_suf__[*i*]_|} be the *reading depth* of suffix *S*_suf__[*i*]_. When searching for matches of Q in *S*, we observe that algorithms *ScanAlign* and *LScanAlign* scan *S* computing dist(Q,S[p..q]) for every window substring of length *q* − *p* + 1 = *m* + *d*. In the suffix array, each substring *S*[*p*..*q*] is represented by a suffix *S*_suf__[*i*]_ up to reading depth *p*_*i*_, i.e. there is a substring *S*_suf__[*i*]_[1..*p*_*i*_] such that *S*_suf__[*i*]_[1..*p*_*i*_] = *S*[*p*..*q*]. To match Q in *S* using a suffix array, we simulate a depth first traversal of the lcp interval tree [30] of *S* on the enhanced suffix array of *S* such that the reading depth of each suffix is limited by *p*_*i*_. That is, we traverse the suffix array of *S* top down, computing the sequence-structure edit distance $\text{dist}(Q,{S_{\mathrm{suf}}}_{\left[i\right]}[1\mathrm{..}p_{i}])$for each suffix *S*_suf__[*i*]_. We recall that candidate matches of Q have length between *m* − *d* and *m* + *d* and that *p*_*i*_ ≤ *m* + *d*. In case *p*_*i*_ < *m* − *d*, we can skip *S*_suf__[*i*]_. Also, remember that all candidate matches shorter than *p*_*i*_ are obtained as a by-product of the computation of $\text{dist}(Q,{S_{\text{suf}}}_{\left[i\right]}[1\mathrm{..}p_{i}])$. Hence, for every *p*^′^, *m* − *d* ≤ *p*^′^ ≤ *p*_*i*_, if $\text{dist}(Q,{S_{\text{suf}}}_{\left[i\right]}[1\mathrm{..}p^{\prime }])\leq K$ we report [suf[*i*]..suf[*i*] + *p*^′^] as a matching interval of Q in *S*. That is, Q matches substring *S*[suf[*i*]..suf[*i*] + *p*^′^] beginning at position suf[*i*] of *S*.

**Figure 5:**
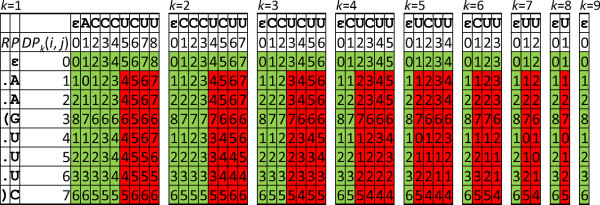
***DP* tables for the sequence-structure alignment computation of RSSP**Q=(AAGUUUC,..(...))** and substring*S***_**suf**__**[*i*]**_ **[1..8] = ACCCUCUU.** Given that suffix *S*_suf__[*i*]_ shares a common prefix of length lcp[*i*] = 4 with *S*_suf__[*i*−1]_, algorithm *LESAAlign* reuses the entries in green and computes the entries in red. Used operation costs: *ω*_d_ = *ω*_m_ = 1, *ω*_b_ = *ω*_a_ = 2, and *ω*_r_= 3.

Our algorithm for the suffix array traversal and $\text{dist}(Q,{S_{\mathrm{suf}}}_{\left[i\right]}[1\mathrm{..}p_{i}])$ computation, hereinafter called *LESAAlign*, builds on algorithms *ScanAlign* and *LScanAlign*. *ScanAlign* and *LScanAlign* exploit overlapping substrings of consecutive window substrings to avoid recomputation of *DP* matrices entries. *LESAAlign* exploits the enhanced suffix array in two different ways. First, for a single suffix *S*_suf__[*i*]_, *i* > 0, it benefits from the common prefix of length lcp[*i*] between two consecutive suffixes *S*_suf__[*i*]_ and *S*_suf__[*i*−1]_ by avoiding the recomputation of columns *j*, 1 ≤ *j* ≤ lcp[*i*] − *k* + 1, of each matrix *DP*_*k*_. This means that, for *lcp* = min{*p*_*i*_,lcp[*i*]}, it avoids the recomputation of $\overset{\text{lcp}}{\underset{k=1}{\sum }}\text{lcp}-k+1$ columns for *S*_suf__[*i*]_. See an example in Figure 5. We observe that if *p*_*i*_ ≤ *lcp*, no *DP* entry needs to be recomputed. In this case, two situations arise: 

1. If *p*_*i*_ ≤ *lcp* and $\text{dist}(Q,{S_{\mathrm{suf}}}_{\left[i-1\right]}[1\mathrm{..}p_{i-1}])\leq K$, then clearly $\text{dist}(Q,{S_{\mathrm{suf}}}_{\left[i\right]}[1\mathrm{..}p_{i}])\leq K$ and at least one match of Q starts at position suf[*i*] of *S*; and

2. If *p*_*i*_ ≤ *lcp* and $\text{dist}(Q,{S_{\mathrm{suf}}}_{\left[i-1\right]}[1\mathrm{..}p_{i-1}])>K$, then $\text{dist}(Q,{S_{\mathrm{suf}}}_{\left[i\right]}[1\mathrm{..}p_{i}])>K$.

These situations allow *LESAAlign* to benefit from the enhanced suffix array in a second important way. That is, it skips all suffixes *S*_suf__[*i*]_, *S*_suf__[*i*+1]_,..., *S*_suf__[*j*]_ sharing a common prefix of at least length *lcp* with *S*_suf__[*i*−1]_. To find the index *j* of the last suffix *S*_suf__[*j*]_ to be skipped, it suffices to look for the largest *j* such that min{lcp[*i*],lcp[*i*+1],...,lcp[*j*]} ≥ *lcp*. If the first situation above holds, there are matches of Q in *S* at positions suf[*i*], suf[*i* +1 ],..., suf[*j*]. We note that suffixes can also be efficiently skipped using so-called skip-tables as described in [36]. However, to save the 4*n* additional bytes required to store such tables we do not use them here. Our algorithm continues the top-down traversal of the suffix array with suffix *S*_suf__[*j*+1]_, taking into account that the *DP* tables were last computed for *S*_suf__[*i*−1]_. Consequently, the length of the longest common prefix between *S*_suf__[*i*−1]_ and *S*_suf__[*j*+1]_ to be considered in the processing of *S*_suf__[*j*+1]_ is min{lcp[*i*],lcp[*i*+1],...,lcp[*j*],lcp[*j*+1]}.

We also incorporate in our index-based algorithm the early-stop alignment computation scheme of algorithm *LScanAlign*. This allows to skip suffixes *S*_suf_[*i*] as soon as it becomes clear that the sequence-structure edit distance of RSSP Q and *S*_suf__[*i*]_ up to reading depth *p*_*i*_ will exceed the cost threshold K. For this, *LESAAlign* progressively aligns regions of Q to a substring of the current suffix as in algorithm *LScanAlign*, checking whether the cost of each subalignment remains below the cost threshold K, thus applying Lemma 2. If the cost exceeds K, the alignment computation of the remaining pattern regions is skipped and the algorithm proceeds with processing the next suffix. To avoid recomputing as many entries of the *DP* matrices as possible while traversing the suffix array, *LESAAlign* differs from *LScanAlign* in the way it manages (non-) aligned regions for each suffix. Lemma 1, which algorithm *LScanAlign* applies to support early-stop computation, relies on scanning the searched RNA sequence *S* and overlapping window substrings. This makes it unsuitable for use with the suffix array. Instead, *LESAAlign* only uses information from the lcp table as follows. Let *z* be the number of regions of Q indexed from 1 to *z* and *T* = *S*_suf__[*i*]_[1..*p*_*i*_] be the current substring. When progressively aligning the regions of Q to a substring of *T*, we store the index *r* of the first region whose alignment cost exceeds K, if there is any. That is, for the first region Q[x..y] whose index *r* we store, it holds that for every *d*^′^, −*d* ≤ *d*^′^ ≤ min{*d*,*x*}, $\text{dist}(Q[\text{x..y}],T_{x+d^{\prime }}[1\mathrm{..l}+z])>k$ with *l* = *y* − *x* + 1, *z* ∈ {|*d*^′^|−*d*,−|*d*^′^|+*d*}, and *y*+*d*^′^ ≤ *m* + *d* (see Lemma 2). Then, when aligning Q to a subsequent substring *S*_suf__[*j*]_[1..*p*_*j*_], we must distinguish the regions of Q*previously computed* from regions *not computed*. 

• *Previously computed pattern regions* are all regions whose index is strictly smaller than *r*. The alignment computation of these regions profits from the common prefix between *S*_suf__[*i*]_[1..*p*_*i*_] and *S*_suf__[ *j*]_[1..*p*_*j*_] by avoiding the recomputation of *DP* matrices columns as described above.

• *Non-computed pattern regions* are all regions whose index is larger than or equal to *r*. In this case, all *DP* matrices columns of the respective pattern region need to be computed, even if *S*_suf__[*i*]_[1..*p*_*i*_] and *S*_suf__[ *j*]_[1..*p*_*j*_] share a common prefix.

We observe that longer ranges of suffixes not containing matches to Qcan be skipped thanks to the early-stop alignment computation scheme. Note that the left-most character of *T* needed to assert $\text{dist}(Q[\text{x..y}],T_{x+d^{\prime }}[1\mathrm{..l}+z])>K$ is *T*[*x* + *l* + *d* − 1] = *T*[*x* + *y* − *x* + 1 + *d* − 1] = *T*[*y* + *d*] as *l* = *y* − *x* + 1. Therefore, no suffix sharing prefix *T*[1..*y* + *d*] can match Q and thus can be skipped in the top-down traversal of the suffix array of *S*. Because in most cases *y* + *d* < *p*_*i*_, more suffixes are likely to share a prefix of length *y* + *d* than of length *p*_*i*_ with *S*_suf__[*i*]_. For the pseudocode of algorithm *LESAAlign*, see Section 1 of Additional file 1.

### Enhanced index-based search: LGSlinkAlign

Given an RSSP Q to be searched in an RNA sequence *S*, algorithm *LESAAlign* is very fast when it can 

• avoid recomputation of *DP* matrices columns due to a common prefix between suffixes of *S*; and

• skip long ranges of suffixes of the suffix array suf whose common prefix up to a required reading depth are known to match or not match Q.

Therefore, *LESAAlign* exploits repetitions of substrings of *S*, i.e. substrings shared by different suffixes, and information of the lcp table to save computation time. However, the use of information of the lcp table alone does not necessarily lead to large speedups. Consider e.g. the *DP* matrices for the computation of the alignment of Q=(AAGUUUC,..(...)) and substring *S*_suf__[4]_[1..*p*_4_] = ACCCUCUU in Figure 5. The enhanced suffix array of *S* is shown in Figure 4. The substring *S*_suf__[4]_[1..*p*_4_] of length 8 shares a common prefix of length lcp[4] = 4 with the previously processed substring *S*_suf__[3]_[1..*p*_3_]. Despite this common prefix, still 182/252 ≈ 72*%* of the *DP* matrices entries need to be computed (disregarding initialization rows and columns 0) in case no early-stop is possible, i.e. in case K>4. This is more than the at most 56/252 ≈ 22*%* of the *DP* matrices entries computed by the online algorithm *LScanAlign* for a window shift.

Our next goal is to develop an algorithm traversing the enhanced suffix array of *S* that: 

1. can skip more suffixes; and

2. improves the use of already computed *DP* matrices entries, reusing computed entries for as many suffixes as possible.

To address the first goal, we motivate our method by recalling the alignment computation example in Figure 2. In this example, one of the regions of Q=(AAGUUUC,..(…)) is Q[3..7]=(GUUUC,(…)). Assume K=d=1 and observe that $\text{dist}(Q[3\mathrm{..}7],T_{3+d^{\prime }}[1\mathrm{..}5+z])>1$ for every *d*^′^, −1 ≤ *d*^′^ ≤ 1, *z* ∈ {|*d*^′^|−1,−|*d*^′^| + 1}, i.e. the alignment cost for this pattern region already exceeds the cost threshold of 1 (in accordance with Lemma 2). In other words, Q[3..7] cannot align to any of the substrings *T*[2..6] = CCCUC, *T*[3..6] = CCUC, *T*[3..7] = CCUCU, *T*[3..8] = CCUCUU, or *T*[4..8] = CUCUU with a cost lower than 1. Observe further that the alignment computation of region Q[3..7] does not depend on any previous computation of any other region. We can therefore conclude that no suffix containing substring *T*[2..8]=CCCUCUU from position 2 to 8 can match Q, independently of any prefix of length 1. Our goal is to find and eliminate from the search space all such suffixes, in addition to skipping all suffixes sharing prefix *T*[1..8] as performed by *LESAAlign*. That is, we want to skip suffixes sharing a substring, not limited to a prefix, whose alignment cost to a pattern region exceeds cost threshold K.

Let *S* be an arbitrary RNA sequence and *T*[*x*..*y*] = *S*_suf__[*i*]_[*x*..*y*] contain all substrings whose alignment cost to a region of an RSSP Q exceeds threshold K. Consider the following two cases for skipping suffixes that cannot match Q as a consequence of containing substring *T*[*x*..*y*] from position *x* to *y*. (1) For any value of *x*, all suffixes sharing prefix *T*[1..*y*] can be skipped as performed by algorithm *LESAAlign*. (2) Now let *x* > 1. To find all suffixes of *S* sharing substring *T*[*x*..*y*] from position *x* to *y*, we first locate all suffixes sharing *T*[*x*..*y*] as a prefix. We begin by locating one such suffix, in particular the suffix of index suf[*j*] that contains all but the first *x*^′^ = *x* − 1 characters of *S*_suf__[*i*]_, i.e. suffix ${S_{\mathrm{suf}}}_{\left[j\right]}={S_{\mathrm{suf}}}_{[i]+x^{\prime }}$. We determine *j* using a generalization of a concept originated from suffix trees. It is a property of suffix trees that for any internal node spelling out string *T* there is also an internal node spelling out *T*_2_ whenever |*T*| > 1 [37]. A pointer from the former to the latter node is called a *suffix link*. In the case of suffix arrays, a suffix link can be computed using the inverse suffix array suf^−1^ of *S*$. suf^−1^ is a table in the range 1 to *n* + 1 such that suf^−1^[suf[*i*]] = *i*. It requires 4*n* bytes and can be computed via a single scan of suf in *O*(*n*) time. Given table suf^−1^, we can define the suffix link from *T* = *S*_suf__[*i*]_ to *T*_2_ = *S*_suf__[*i*]+1_ as *link* = suf^−1^[suf[*i*] + 1], i.e. it holds that suf[*link*] = suf[*i*] + 1. Now, if *x*^′^ = 1, we already find that the index suf[*j*] of the suffix containing all but the first character of *S*_suf__[*i*]_ is suf[*j*] = suf[*link*] because ${S_{\mathrm{suf}}}_{\left[\text{link}\right]}={S_{\mathrm{suf}}}_{[i]+x^{\prime }}$ holds. However, we also want to be able to determine *j* for any *x*^′^ ≥ 1. The obvious solution is to compute suffix links *x*^′^ successive times. Each suffix link skips the first character of the previously located suffix. For a more efficient solution, we generalize suffix links to point directly to the suffix without a prefix of any length *x*^′^ of the initial suffix. For this purpose we define a function link:N×N→N as:

(10) $$\text{link}(i,x^{\prime })={\text{suf}}^{-1}[\text{suf}[i]+x^{\prime }].$$

Then, by letting *j* = *link*(*i*,*x*^′^), ${S_{\mathrm{suf}}}_{\left[\text{link}(i,x^{\prime })\right]}={S_{\mathrm{suf}}}_{[i]+x^{\prime }}$ holds for any *x*^′^ ≥ 1. All suffixes sharing *T*[*x*..*y*] as a prefix are all suffixes in the range *j*_start_ to *j*_end_ where *j*_start_ is the smallest and *j*_end_ is the largest index satisfying min{lcp[*j*_start_ + 1],...,lcp[*j*],...,lcp[*j*_end_]} ≥ *y* − *x* + 1. Finally, we find that all suffixes of *S* sharing substring *T*[*x*..*y*] from position *x* to *y* are all ${S_{\mathrm{suf}}}_{[j^{\prime }]-x^{\prime }}$, $j_{\text{start}}\leq j^{\prime }\leq j_{\text{end}}$, satisfying $\mathrm{suf}[j^{\prime }]>x^{\prime }$. To skip these suffixes not containing matches to Q in the top-down traversal of the suffix array suf, we mark their positions as true (for already“processed”) in a bit array vtab of *n* bits. The suffix array traversal proceeds from position suf[*i*], but skips the marked suffixes when their positions are reached.

We remark that the described method for skipping suffixes can profit from a resorting according to the order by which RSSP regions are aligned. In the alignment computation example in Figure 2, determining $\text{dist}(Q[3\mathrm{..}4],T_{3+d^{\prime }}[1\mathrm{..}2+z])>1$, −1≤*d*^′^ ≤ 1, *z* ∈ {|*d*^′^|−1,−|*d*^′^| + 1}, does not depend on character *T*[1] and region Q[1..1]. Hence, region Q[1..1] is unnecessarily aligned first when the regions are sorted by a top-down analysis of the *DP* tables. To decrease the chance that unnecessary computations occur, we sort the *RSSP* regions to begin aligning with the left-most *RSSP* region Q[x..y] not depending on the alignment of any other region and satisfying *x* − *d* > 1.

We now address the second goal, namely reusing computed *DP* matrices entries for as many suffixes as possible. Recall that computing the sequence-structure edit distance $\text{dist}(Q,{S_{\mathrm{suf}}}_{\left[i\right]}[1\mathrm{..}p_{i}])$ for each suffix *S*_suf__[*i*]_ up to reading depth *p*_*i*_ means computing *p*_*i*_ + 1*DP* matrices, one for each suffix *T*_*k*_ of string *T* = *S*_suf__[*i*]_[1..*p*_*i*_], 1 ≤ *k* ≤ *m*^′^, and one for the empty sequence *ε*. Observe that each suffix *T*_*k*_, *T*_*k*_ ≠ *T*, also occurs itself as a prefix of a suffix in table suf, i.e. there exists a suffix *S*_suf__[*j*]_ shorter than *S*_suf__[*i*]_ by exactly *k* − 1 characters which has prefix *T*_*k*_. Consequently, *T*_*k*_ is processed again in an alignment to RSSP Q at a different point in time during the traversal of suf. Let *T*^′^ = *S*_suf__[*j*]_[1..*p*_*j*_]. Now note that if *T*^′^ is at a (nearly) contiguous position in suf to *T*, *T*^′^ and *T* are likely to share a common prefix due to their similar lexicographic ranking. This allows algorithm *LESAAlign* to avoid recomputation of *DP* matrices columns by using information from the lcp table. Unfortunately, *T*^′^ and *T* can be lexicographically ranked far away from each other in table suf, meaning that the *DP* matrices computed for *T*^′^ either: 

• were already computed once because *T*^′^ is lexicographically smaller than *T*, but were discarded to allow the processing of other suffixes until *T* was traversed; or

• are computed for the first time otherwise, but will not be reused to also allow the processing of other suffixes until *T*^′^ occurs in table suf as a prefix of a suffix itself.

In both cases, redundant computations occur. To avoid this, we optimize the use of computed *DP* matrices by processing *T*^′^ directly after processing *T* for fixed *k* = 2, recalling that *T* = *S*_suf__[*i*]_[1..*p*_*i*_] and *T*^′^ = *S*_suf__[*j*]_[1..*p*_*j*_]. This value of *k* implies that *S*_suf__[*j*]_ does not contain the first character of *S*_suf__[*i*]_ and that we can locate *S*_suf__[*j*]_ in table suf by computing the suffix link *j* = *link*(*i*,1). Also, *k* = 2 implies that *T*^′^ only differs by its last character from *T*, aside from not beginning with character *T*[1]. Therefore, to determine $\text{dist}(Q,T^{\prime })$, we only have to compute the last column of the *DP* matrices required to compute dist(Q,T) as shown by Lemma 1. We note that, because *i* and *j* are not necessarily contiguous positions in suf, we mark the processed suffix *S*_suf__[*j*]_ in the bit array vtab so that it is only processed once. If no match to RSSP Q begins at position suf[*j*], we also mark and skip every suffix sharing the substring with *T*^′^ whose alignment to a region of Q is known to exceed threshold K. Once *T*^′^ is processed and all possible suffixes are skipped, we recursively repeat this optimization scheme by setting *T* = *T*^′^ and processing the next $T^{\prime }={S_{\mathrm{suf}}}_{\left[j^{\prime }\right]}[1\mathrm{..}p_{j^{\prime }}]$ where *j*^′^ = *link*(*j*,1). The recursion stops when $p_{j^{\prime }}<m-d$, meaning that *T*^′^ is too short to match Q, or when suf[*j*^′^] is already marked as processed in vtab. The suffix array traversal proceeds at position *i* + 1 repeating the entire scheme.

We call our algorithm incorporating the presented improvements *LGSlinkAlign*. For its pseudocode, see Section 1 of Additional file 1. *LGSlinkAlign* inherits all the improvements of the above presented algorithms. In summary, its improvements are as follows. 

• *LGSlinkAlign* traverses the enhanced suffix array of the searched sequence *S*, i.e. the suffix array suf enhanced with tables lcp and suf^−1^. During this traversal, it benefits from common prefixes shared among suffixes to (1) avoid the computation of *DP* matrix columns and to (2) skip ranges of suffixes known to match or not match RSSP Q as in algorithm *LESAAlign*.

• The suffix array traversal is predominantly top down, but non-contiguous suffixes are processed to optimize the use of computed *DP* matrices.

• *LGSlinkAlign* stops the alignment computation as early as the alignment cost of a region of RSSP Q and a substring of the prefix of the current suffix exceeds threshold K, an improvement first introduced in algorithm *LScanAlign*.

• Due to the early-stop computation scheme, suffixes sharing common prefixes shorter than *m* + *d* can be skipped, leading to larger ranges of skipped suffixes. The early-stop computation scheme also helps to identify and skip non-contiguous suffixes sharing a common substring which is not their prefix.

#### Example: searching for an RSSP with algorithm LGSlinkAlign

We elucidate the ideas of algorithm *LGSlinkAlign* with the following example. Consider the RSSP Q=(AAGUUUC,..(...)) to be matched in the sequence *S* whose enhanced suffix array is shown in Figure 4. To keep the example simple, we only allow a small cost threshold and number of indels, i.e. we set K=d=1. The costs of the edit operations are *ω*_d_ = *ω*_m_ = *ω*_b_ = *ω*_a_ = 1 and *ω*_r_ = 2. When traversing the enhanced suffix array of *S*, *LGSlinkAlign* always begins to align Q to a substring of *S* with region Q[4..6], because the alignment computation of this region does not depend on any other region. In addition, the left index of this region satisfies 4 − *d* > 1. This means that the alignment computation of region Q[1..2] is avoided if the cost of aligning region Q[4..6] exceeds the threshold K. The algorithm starts the traversal of the enhanced suffix array of *S* aligning Q[4..6] to substrings of *T* = *S*_suf__[1]_[1..*p*_1_] = *S*_14_[1..8] from positions 4 − *d* = 3 and 6 + *d* = 7. For this, it computes $\text{dist}(Q[4\mathrm{..}6],T_{4+d^{\prime }}[1\mathrm{..}3+z])$ for −1 ≤ *d*^′^ ≤ 1 and *z* ∈ {|*d*^′^|−1,−|*d*^′^|+1}. Observe that $\text{dist}(Q[4\mathrm{..}5],T_{4+d^{\prime }}[1\mathrm{..}2+z])>1$ holds. Hence (1) no suffix with prefix *T*[1..6] = AACACC can match Q and thus can be skipped and (2) no suffix containing substring *T*[3..6] = CACC from position 4−*d* = 3 to 5 + *d* = 6 can match Q and thus can be skipped as well. We notice that there is no other suffix with prefix AACACC because lcp[2] < 6, so we analyze case (2). The algorithm looks for suffixes sharing substring CACC from position 3 to 6. It begins by locating suffixes without the first two characters of *T* and containing CACC as a prefix. It follows the suffix link *link*(1,2) = suf^−1^[suf[1]+2] = suf^−1^[16] = 7 and looks for the smallest *j*_start_ and largest *j*_end_ satisfying min{lcp[*j*_start_ + 1],...,lcp[8],...,lcp[*j*_end_]} ≥ 4 = |CACC|. It finds that *j*_start_ = 5 and *j*_end_ = 8, since min{lcp[5+1],lcp[7],lcp[8]} = min{4,5,5} ≥ 4 holds. The suffixes containing CACC from position 3 to 6 are *S*_suf__[5]−2_ = *S*_11_, *S*_suf__[6]−2_ = *S*_7_, and *S*_suf__[8]−2_ = *S*_14_. *S*_11_and *S*_7_ are marked in the bit array vtab, whereas *S*_14_ = *S*_suf__[1]_ was already processed and does not need to be marked. We observe that *S*_suf__[7]−2_ = *S*_−1_ is not a valid suffix. To reuse as many computed *DP* matrices entries as possible, the algorithm next processes the suffix *S*_suf__[*j*]_ which does not contain the first character of *S*_suf__[1]_. It determines *j* = *link*(1,1)=suf^−1^[suf[1]+1] = 11 and sets *T* = *S*_suf__[12]_[1..*p*_12_] = *S*_15_[1..8]. The alignment to this substring *T* begins with its substrings from positions 3 to 7 and Q[4..6]. We observe that $\text{dist}(Q[4\mathrm{..}5],T_{4+d^{\prime }}[1\mathrm{..}2+z])>1$ holds and consequently *T* cannot match Q. Because suffix *S*_suf__[12]_ = *S*_15_ was traversed via a suffix link, it is marked as processed in vtab. We now again analyze two cases of suffixes that cannot match Q and therefore can be skipped: (1) suffixes sharing prefix *T*[1..6] = CCACCC and (2) suffixes containing substring *T*[3..6] = ACCC from position 3 to 6. Satisfying case (1) are suffixes *S*_suf__[11]_ = *S*_1_ and *S*_suf__[10]_ = *S*_8_ since lcp[12] ≥ 6 and lcp[11] ≥ 6. These suffixes are marked in vtab. We now check if there are suffixes satisfying case (2). The algorithm begins by locating suffixes containing substring *T*[3..6] = ACCC as a prefix. For this, it follows the suffix link *link*(12,2) = suf^−1^[suf[12] + 2] = 4 and determines *j*_start_ = 2 and *j*_end_ = 4. The property min{lcp[2 + 1],lcp[4]} ≥ 4 is satisfied. The suffixes containing ACCC from position 3 to 6 are *S*_suf__[2]−2_ = *S*_8_, *S*_suf__[3]−2_ = *S*_1_, and *S*_suf__[4]−2_ = *S*_15_. Since these were already marked in vtab, none of them needs to be marked. The algorithmic scheme of *LGSlinkAlign* to reuse as many computed *DP* matrices entries as possible continues processing other suffixes which are located by iteratively following the suffix links. It locates suffixes *S*_suf__[8]_, *S*_suf__[4]_, *S*_suf__[18]_, and *S*_suf__[19]_ because *link*(12,1) = 8, *link*(8,1) = 4, *link*(4,1) = 18, and *link*(18,1) = 19, respectively. These suffixes are processed analogously as above, one after the other, not resulting in matches to Q. The iteration then leads to suffix *S*_suf__[20]_, since *link*(19,1) = 20. However, |*S*_suf__[20]_| < *m*−*d*, meaning that this suffix is too short to contain a match to Q. This causes the iteration to stop. The suffix array traversal proceeds and repeats the entire matching scheme from the suffix that follows the last processed suffix not located via a suffix link, i.e. suffix *S*_suf__[2]_. After processing and skipping all possible suffixes, we note that *LGSlinkAlign* does not report any matches for the defined cost threshold and allowed number of indels K=d=1. By setting K=5, it reports a match at position 16.

### RNA secondary structure descriptors based on multiple ordered RSSPs

RNAs with complex branching structures often cannot be adequately described by a single RSSP due to difficulties in balancing sensitivity, specificity, and reasonable running time of the used search algorithm. Although their description by a single short RSSP specifying an unbranched fragment of the molecule might be very sensitive, it is often too unspecific and likely to generate many spurious matches when searching for structural homologs in large sequence databases or complete genomes. In contrast, using a single long RSSP often requires a higher cost threshold K for being sensitive enough which in turn, together with the increased RSSP length, has a negative influence on the search time. This might lead to disadvantageous running times in larger search scenarios in practice.

We solve this problem by applying the powerful concept of RNA secondary structure descriptors (SSDs for short) recently introduced in [23]. The underlying concept of SSDs is similar to the idea of PSSM family models [38], which are successfully used for fast and sensitive protein homology search. SSDs use the information of multiple ordered RSSPs derived from the decomposition of an RNA’s secondary structure into stem-loop like structural elements. In a first step, approximate matches to the single RSSPs the SSD consists of are obtained using one of the algorithms presented above. From these matches, either local or global high-scoring chains are computed with the efficient chaining algorithms described in [23]. These algorithms take the chain’s score, i.e. the weights of the fragments in the chain, into account (see [23] for details). For chaining of approximate RSSP matches, we use the fragment weight $\omega_{Q}^{*}-\text{dist}(Q,T)$ for an RSSP Q of length *m* matching substring *T*, where $\omega_{Q}^{*}=m*\omega_{m}+\text{bps}*\omega_{r}$ and *bps* denotes the number of base pairs in Q. Here $\omega_{Q}^{*}$ is the maximal possible weighting Q can gain when being aligned and therefore it reflects the situation of a perfect match between Q and *T*. With this definition of a fragment’s weight, a positive weight is always guaranteed, thus satisfying a requirement for the chaining algorithm. Once the chaining of matches to the RSSPs is completed, the high-scoring chains are reported in descending order of their chain score. By restricting to high-scoring chains, spurious RSSP matches are effectively eliminated. Moreover, the relatively short RSSPs used in an SSD can be matched efficiently with the presented algorithms leading to short running times that even allow for the large scale application of approximate RSSP search.

## Results and discussion

### Implementation and computational results

We implemented (1) the fast index-based algorithms *LESAAlign* and *LGSlinkAlign*, (2) the online algorithms *LScanAlign*, *ScanAlign*, both operating on the plain sequence, and (3) the efficient global and local chaining algorithms described in [23]. In our experiments we use *ScanAlign*, which is the scanning version of the method proposed in [25], for reference benchmarking. All algorithms are included in the program *RaligNAtor*. The algorithms for index construction were implemented in the program *sufconstruct*, which makes use of routines from the *libdivsufsort2* library (see http://code.google.com/p/libdivsufsort/) for computing the suf table in *O*(*n* log *n*) time. For the construction of table lcp we employ our own implementation of the linear time algorithm of [35]. All programs were written in C and compiled with the GNU C compiler (version 4.5.0, optimization option -O3). All measurements are performed on a Quad Core Xeon E5620 CPU running at 2.4 GHz, with 64 GB main memory (using only one CPU core). To minimize the influence of disk subsystem performance, the reported running times are user times averaged over 10 runs. Allowed base pairs are canonical Watson-Crick and wobble, unless stated otherwise. The used sequence-structure operation costs are *ω*_d_ = *ω*_m_ = *ω*_b_ = *ω*_a_ = 1 and *ω*_r_ = 2.

#### Comparison of running times

**Figure 6:**
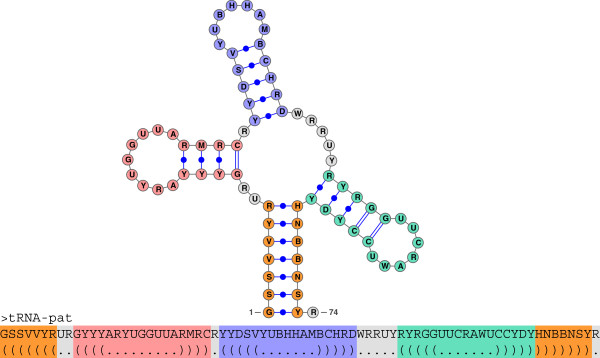
**Consensus secondary structure of the tRNA family (Acc.: RF00005) as drawn by*VARNA***[39]** (top) and respective sequence-structure pattern** **tRNA-pat****(bottom).**

**Figure 7:**
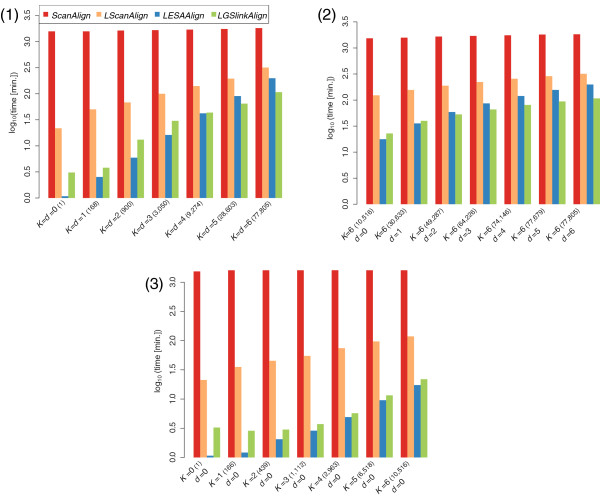
**Running times (in minutes and** **log**_**10**_** scale) needed by the different algorithms to search with an RSSP describing the tRNA in** **RFAM10.1****.** In (1) the cost threshold K and the number of allowed indels *d* are identical. In (2) K=6is constant and *d* ranges from 0 to 6. In (3) *d* = 0 is constant and K ranges from 0 to 6. The numbers of resulting matches are given on the x-axes in brackets.

In a first benchmark experiment we measure the running times needed by the four algorithms to search with a single RSSP under different cost thresholds K and number of allowed indels *d*. We set (1) K=d varying the values in the interval [0,6], (2) K=6varying *d* in the interval [0,6], and (3) *d* = 0 varying K in the interval [0,6]. The searched dataset contains 2,756,313 sequences with a total length of ≈786 MB from the full alignments of all Rfam release 10.1 families. The construction of all necessary index tables needed for *LESAAlign* and *LGSlinkAlign* with *sufconstruct* and their storage on disk required 372 seconds. In the following we refer to this dataset as RFAM10.1 for short. In this experiment we use the RSSP tRNA-pat of length *m* = 74 shown in Figure 6 describing the consensus secondary structure of the tRNA family (Acc.: RF00005). The results of this experiment are presented in Figure 7 and Table S4, S5, and S6 of Additional file 1. *LGSlinkAlign* and *LESAAlign* are the fastest algorithms. *LGSlinkAlign* is faster in particular for increasing values of K and *d*, being only slower than *LESAAlign* for small values of K and *d* and for fixed *d* = 0. The advantage of *LGSlinkAlign* over *LESAAlign* with higher values of K and *d* is explained by the increased reading depth in the suffix array implicated by K and *d* and the fewer suffixes sharing a common prefix that can be skipped. This holds for both *LGSlinkAlign* and *LESAAlign*, however *LGSlinkAlign* counterbalances this effect by reusing computed *DP* matrices for non-contiguous suffixes of the suffix array. In a comparison to the two online algorithms considering only approximate matching, i.e. K≥1, the speedup factor of *LGSlinkAlign* over *ScanAlign* (*LScanAlign*) is in the range from 560 for K=1 and *d* = 0 to 17 for K=d=6 (from 15 for K=2 and *d* = 0 to 3 for K=d=6). *LESAAlign* achieves a speedup factor over *ScanAlign* (*LScanAlign*) in the range from 1,323 for K=1 and *d* = 0 to 9 for K=d=6 (29 for K=1 and *d* = 0 to 1.6 for K=d=6). In a comparison between the online algorithms, *LScanAlign* is faster than *ScanAlign* by up to factor 45 for K≥1. In summary, all algorithms except *ScanAlign* profit from low values of K and *d* reducing their search times. This is a consequence of the use of the early-stop alignment computation scheme. As shown in Figure 7(2), also the number of allowed indels *d* influences the search time. For an additional experiment investigating the influence of K and *d* on the search time required by the four algorithms, see Section 2 of Additional file 1. A further experiment, described in Section 3 of Additional file 1, compares *RaligNAtor* and the widely used tool *RNAMotif*[15] in terms of sensitivity and specificity in searches for the tRNA-pat depicted in Figure 6.

#### Scaling behavior of the online and index-based algorithms

**Figure 8:**
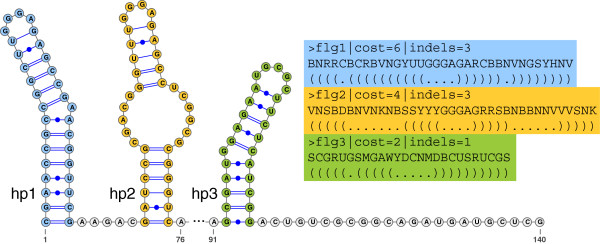
**Consensus secondary structure of family flg-Rhizobiales RNA motif (Acc.: RF01736) showing its three stem-loop substructures** **hp1****,****hp2****, and****hp3****as drawn by** ***VARNA***[39]**.** The secondary structure descriptor (SSD) for this family, on the right-hand side, consists of three RSSPs flg1, flg2, and flg3 derived from the stem-loop substructures.

**Figure 9:**
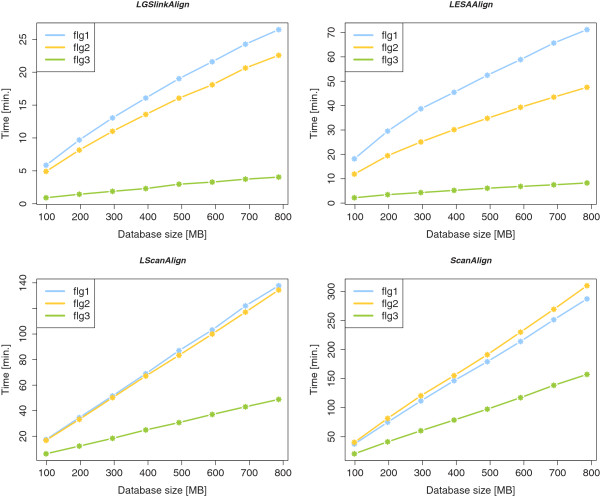
**Scaling behavior of algorithms *LGSlinkAlign *, *LESAAlign *, *LScanAlign *, and *ScanAlign * when searching with RSSPs** **flg1****,****flg2****, and****flg3****, in subsets of** **RFAM10.1****, of different length.** For details, see main text.

In a second experiment we investigate how the search time of algorithms *ScanAlign*, *LScanAlign*, *LESAAlign*, and *LGSlinkAlign* scales on random subsets of RFAM10.1 of increasing size. The searched RSSPs flg1, flg2, and flg3 were derived from the three stem-loop substructures the members of family flg-Rhizobiales RNA motif (Acc.: RF01736) [40] fold into. These patterns differ in length, cost threshold K and number of allowed indels *d*; see Figure 8 for their definition, noting that K and *d* are simply denoted *cost* and *indels* in the *RaligNAtor* RSSP syntax. The results are shown in Figure 9 and Table S7 of Additional file 1. *LGSlinkAlign* and *LESAAlign* show a sublinear scaling behavior, whereas *LScanAlign* and *ScanAlign* scale linearly. The fastest algorithm is *LGSlinkAlign*, requiring only 11.68 (53.08) minutes to search for all three patterns in the smallest (full) subset. The second fastest algorithm is *LESAAlign*, followed by *LScanAlign* and *ScanAlign*, which require 32.27 (126.97), 40.47 (321.01), and 98.35 (754.66) minutes, respectively, to search for all the patterns in the smallest (full) subset. This corresponds to a speedup of 8.4 to 14.2 of *LGSlinkAlign* over *ScanAlign* on the smallest and the full subsets. Comparing the search time for pattern flg3 individually, the speedup of *LGSlinkAlign* over *ScanAlign* ranges from 22.6 to 38.8. We also observe that *ScanAlign* requires the longest time to match the longest pattern flg2 of length *m* = 37. The other algorithms profit from the early-stop computation approach to reduce the search time for this pattern on every database subset.

#### Influence of stem and loop lengths on the search time

**Figure 10:**
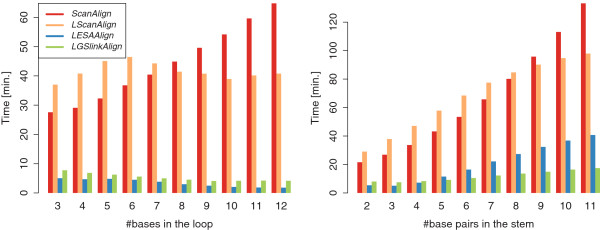
Search times for different number of bases in the loop (left-hand side) and base pairs in the stem (right-hand side) for given RSSPs.

When searching a database for matches of a given pattern, our algorithms compute the required *DP* matrices using recurrences according to two main cases: either a row corresponds to an unpaired or to a paired base of the pattern. To analyze the influence of the used recurrence on the search time of each algorithm, we search RFAM10.1 for artificial stem-loop patterns. Therefore we vary the number of bases in the loop of pattern Q=(NNNACANNN,(((...)))) from 3 to 12 by using As and Cs. Additionally, we vary the number of base pairs in the stem of pattern Q=(NNACANN,((...))) from 2 to 11 by pairs of Ns. Matching the patterns in these two experiments means to increase the use of the *DP* recurrences in Equations (7) and (8), respectively. The cost threshold and the number of allowed indels are fixed at K=d=3. Allowed base pairs are (A, U), (U, A), (C, G), and (G, C). The results are shown in Figure 10. We observe that increasing the number of bases in the loop has little influence and even reduces the running time of the two fastest algorithms *LGSlinkAlign* and *LESAAlign*. This can be explained by the use of the early-stop alignment computation scheme in these algorithms. The reduction of the running time is explained by the fewer matches that need to be processed as the pattern gets longer and more specific. For an increasing number of base pairs in the stem, *LGSlinkAlign* is the least affected algorithm. We also observe that the linear increase in running time of the basic online algorithm *ScanAlign*, caused by an extension of the pattern by one base pair, is similar to the effect of adding two bases in the loop.

#### RNA family classification by global chaining of RSSP matches

In the next experiment we show the effectiveness of global chaining when searching with two SSDs built for Rfam families Cripavirus internal ribosome entry site (Acc.: RF00458) and flg-Rhizobiales RNA motif (Acc.: RF01736) [40]. These two families present only 53% and 69% sequence identity, respectively, much below the average of ∼80*%* of the Rfam 10.1 families. This illustrates the importance of using both sequence and structure information encoded in the SSDs of this experiment. The SSD of family RF01736 comprises three RSSPs, denoted by flg1, flg2, and flg3 in Figure 8, derived from the three stem-loop substructures the members of this family fold into. The SSD of family RF00458 comprises five RSSPs, denoted by ires1, ires2, ires3, ires4, and ires5 in Figure S5 of Additional file 1, where the last four RSSPs describe the stem-loop substructures the members of this family fold into. ires1 describes a moderately conserved strand occurring in these members. Observe also in Figures 8 and S5 the cost threshold K and allowed number of indels *d* used per pattern, remembering that these are denoted *cost* and *indels* in the *RaligNAtor* RSSP syntax.

Searching with the SSD of family RF00458 in RFAM10.1 delivers 16,033,351 matches for ires1, 8,950,417 for ires2, 1,052 for ires3, 112 for ires4, and 1,222,639 for ires5. From these matches, *RaligNAtor* computes high-scoring chains of matches, eliminating spurious matches and resulting in exactly 17 chains. Each chain occurs in one of the 16 sequence members of the family in the full alignment except in sequence AF014388, where two chains with equal score occur. The highest (lowest) chain score is 171 (162). Using *ScanAlign*, *LScanAlign*, *LESAAlign*, and *LGSlinkAlign*, the search for all five RSSPs requires 688.32, 585.59, 186.88, and 92.25 minutes, respectively, whereas chaining requires 13.66 seconds. See Table S8 of Additional file 1 for the time required to match each pattern using the different algorithms.

The same search is performed using the SSD of family RF01736. It results in 4,145 matches for flg1, 68,024 for flg2, and 67 for flg3. Chaining the matches leads to 15 chains occurring each in one of the 15 sequence members of the family in the full alignment. The highest (lowest) chain score is 163 (156). Using *ScanAlign*, *LScanAlign*, *LESAAlign*, and *LGSlinkAlign*, the search for all three RSSPs requires 755.48, 336.69, 133.58, and 52.86 minutes, respectively, whereas chaining requires 0.03 seconds. The time required to match each pattern using each algorithm is reported in Table S9 of Additional file 1.

We also show that the lack of the sequence-structure edit operations supported by *RaligNAtor* deteriorates sensitivity and specificity in the search for sequence members of families RF00458 and RF01736. For this, we report in Section 4 and Table S10 of Additional file 1 results obtained with the *Structator* tool [23]. *Structator* is much faster but, in contrast to *RaligNAtor*, does not support all sequence-structure edit operations.

#### Importance of structural constraints for RNA family classification

**Figure 11:**
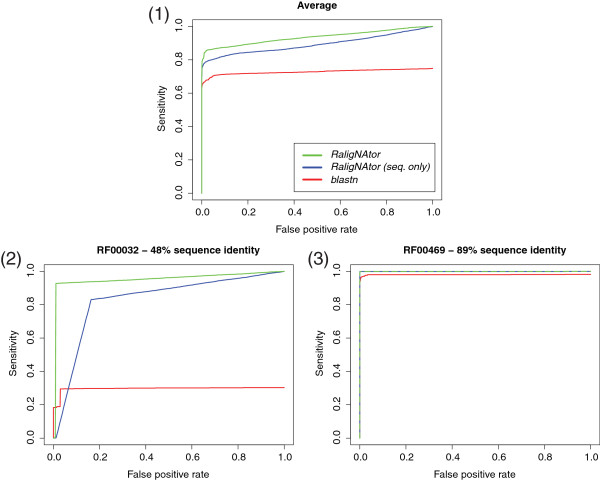
**Results of ROC analyses using *RaligNAtor * with and without base pairing information and *blastn * for the 35 selected Rfam families shown in Table**1. ROC curves showing *RaligNAtor*’s classification performance using (ignoring) base pairing information are shown in green (blue). Blast performance results are shown in red. Subfigure **(1)** shows the performance results averaged over all selected families. **(2)** and **(3)** show each the ROC analysis for the family with the lowest and highest level of sequence identity.

To assess the potential of using RSSPs for reliable RNA homology search on a broader scale and to investigate the effect of using base pairing information, we evaluated *RaligNAtor* on 35 RNA families taken from Rfam 10.1 with different degrees of sequence identity and of different sizes. See Table 1 for more information about the selected families. In our experiment, we compared (1) *RaligNAtor* results obtained by using RSSPs derived from Rfam seed alignments with (2) results obtained for the same RSSPs ignoring base pairing information and (3) results obtained by *blastn*[41] searches with the families’ consensus sequence. For each selected family, we automatically compiled an RSSP Q=(P,R) from the family’s seed alignment using the following procedure: at each position of the RSSP’s sequence pattern *P*, we choose the IUPAC wildcard matching all symbols in the corresponding alignment column. As structure string *R*, we use the secondary structure consensus available in the Rfam seed alignment. From the resulting RSSPs we remove the maximum prefix and suffix containing neither sequence information (i.e. IUPAC symbol N) nor base pairing information. To obtain a query sequence for *blastn*, we compute the consensus sequence from the family’s seed alignment. Because *blastn* does not appropriately handle IUPAC wildcard characters in the query, we choose the most frequent symbol occurring in a column as representative symbol in the consensus sequence. For the *RaligNAtor* searches, we adjust the cost threshold K and number of allowed indels *d* such that we match the complete family. That is, we achieve a sensitivity of 100%. The used operation costs are *ω*_*d*_ = *ω*_*m*_ = 1, *ω*_*b*_ = *ω*_*a*_ = 2, and *ω*_*r*_ = 3. For the Blast searches, we called *blastn* with parameters -m8 -b 250000 -v 250000 and a very relaxed E-value cutoff of 1000. From the two *RaligNAtor* and one *blastn* outputs we count the number of true positives (#TPs) and false positives (#FPs) and compute ROC curves on the basis of the *RaligNAtor* score $\omega_{Q}^{*}-\text{dist}(Q,T)$ and the *blastn* bit score. See Table 1 and Figure 11 for the results of this experiment. A ROC curve with values averaged over all families is shown in Figure 11(1).

**Table 1:** **Results of *RaligNAtor * and *blastn * database searches for members of RNA families of different degrees of sequence identity in**RFAM10.1

					***RaligNAtor***			***RaligNAtor* (sequence only)**					***blastn***			
**Family Acc.**	**Size**	**Seq. ident.**	K=d	**#TP**	**#FP**	**AUC**	**(pAUC)**	K=d	**#TP**	**#FP**	**AUC**	**(pAUC)**	**#TP**	**#FP**	**AUC**	**(pAUC)**
RF00032	9,900	48%	3	9,900	1,088,131	0.95	(0.17)	3	9,900	2,723,135	0.82	(0.09)	3,000	68	0.29	(0.05)
RF00080	688	52%	33	688	698,942	0.71	(0.08)	19	688	1,279,375	0.60	(0.06)	326	540	0.42	(0.06)
RF02003	176	52%	21	176	1,174,167	0.53	(0.03)	6	176	1,168,093	0.32	(0.00)	28	814	0.11	(0.01)
RF00458	16	53%	20	16	88	0.94	(0.18)	14	16	2,688	0.96	(0.18)	12	1,224	0.73	(0.13)
RF00685	131	55%	18	131	40,952	0.98	(0.19)	7	131	103,276	0.97	(0.19)	88	2,945	0.63	(0.10)
RF00167	1,244	56%	25	1,244	2,514,701	0.58	(0.04)	17	1,244	2,611,256	0.28	(0.00)	660	624	0.52	(0.10)
RF01705	598	56%	26	598	2,704,796	0.49	(0.02)	17	598	2,698,712	0.42	(0.00)	57	60	0.08	(0.01)
RF01852	1,050	56%	22	1,050	1,026,233	0.99	(0.19)	14	1,050	1,488,254	0.94	(0.17)	543	83,268	0.44	(0.06)
RF01734	584	57%	10	584	2,614,228	0.69	(0.05)	5	584	2,668,392	0.46	(0.01)	201	114	0.30	(0.05)
RF00556	201	58%	8	201	69,808	0.97	(0.18)	6	201	1,514,311	0.92	(0.15)	91	1,024	0.44	(0.08)
RF00713	14	58%	27	14	10,349	0.99	(0.19)	18	14	16,477	0.88	(0.16)	13	552	0.92	(0.18)
RF00170	41	59%	13	41	53	0.97	(0.18)	9	41	9,197	0.96	(0.18)	29	176	0.70	(0.14)
RF00706	69	59%	13	69	1	1.00	(0.20)	9	69	12	0.97	(0.19)	66	194	0.95	(0.18)
RF00747	29	59%	20	29	130	0.97	(0.18)	16	29	159,898	0.96	(0.18)	28	236	0.96	(0.19)
RF00778	20	59%	33	20	394,560	0.93	(0.17)	23	20	167,029	0.79	(0.13)	17	390	0.84	(0.16)
RF01065	118	59%	17	118	0	1.00	(0.20)	9	118	0	1.00	(0.20)	70	305	0.59	(0.11)
RF01733	9	63%	9	9	0	1.00	(0.20)	7	9	0	1.00	(0.20)	7	918	0.77	(0.15)
RF00522	415	67%	5	415	1,461	0.99	(0.19)	5	415	32,224	0.99	(0.19)	359	391	0.63	(0.10)
RF01862	15	67%	7	15	0	1.00	(0.20)	5	15	0	1.00	(0.20)	10	82	0.66	(0.13)
RF00104	406	69%	24	406	989,362	0.99	(0.19)	14	406	1,560,674	0.99	(0.19)	237	72	0.45	(0.07)
RF00165	431	69%	9	431	0	1.00	(0.20)	8	431	1	0.99	(0.19)	318	192	0.73	(0.14)
RF01185	108	69%	13	108	24,759	0.99	(0.19)	13	108	24,759	0.99	(0.19)	104	329	0.93	(0.18)
RF01838	77	69%	4	77	0	1.00	(0.20)	4	77	0	1.00	(0.20)	77	172	1.00	(0.20)
RF02031	164	71%	17	164	297,941	0.99	(0.19)	12	164	521,018	0.99	(0.19)	100	218	0.60	(0.11)
RF00052	210	72%	16	210	0	1.00	(0.20)	12	210	0	1.00	(0.20)	207	12,496	0.98	(0.19)
RF00543	103	73%	26	103	0	1.00	(0.20)	19	103	0	1.00	(0.20)	102	110	0.99	(0.19)
RF01744	14	73%	7	14	0	1.00	(0.20)	5	14	0	1.00	(0.20)	11	5,377	0.74	(0.14)
RF01769	149	75%	16	149	0	1.00	(0.20)	10	149	0	1.00	(0.20)	149	150	0.99	(0.19)
RF00110	161	81%	19	161	0	1.00	(0.20)	17	161	0	1.00	(0.20)	160	791	0.99	(0.19)
RF01967	50	84%	37	50	660,130	0.98	(0.19)	26	50	475,242	0.98	(0.19)	48	691	0.95	(0.19)
RF01472	26	85%	6	26	0	1.00	(0.20)	1	26	0	1.00	(0.20)	26	412	1.00	(0.20)
RF01953	46	85%	32	46	0	1.00	(0.20)	22	46	0	1.00	(0.20)	46	772	1.00	(0.20)
RF00372	45	86%	28	45	0	1.00	(0.20)	24	45	0	1.00	(0.20)	45	197	0.99	(0.19)
RF01980	43	86%	39	43	830,971	0.97	(0.19)	28	43	702,352	0.96	(0.19)	43	341	1.00	(0.20)
RF00469	1,366	89%	12	1,366	46,351	0.99	(0.19)	7	1,366	99,045	0.99	(0.19)	1,341	474	0.97	(0.19)
Average		66%				0.93	(0.17)				0.89	(0.16)			0.72	(0.14)

Searches are performed using *RaligNAtor* with and without base pairing information (column “*RaligNAtor* (sequence only)”) and using program *blastn* with the families’ seed alignment consensus sequence as query. Column “size” indicates the number of members in a family. Column “seq. ident.” gives the families’ sequence identity as listed in the Rfam database. #TP and #FP stand for number of found true and false positives, respectively. AUC is the area under the curve of the corresponding ROC curves shown in Figures 11, S7, and S8 of Additional file 1. Column pAUC gives the partial area under the curve up to a false positive rate of 20%. For additional details, see main text.

In addition, we show in Figures 11(2) and (3) the results of the ROC analysis for the families with the lowest and highest degree of sequence identity. For the ROC curve of each selected family, see Figures S7 and S8 of Additional file 1. Clearly, by using base pairing information, *RaligNAtor* achieves a higher sensitivity with a reduced false positive rate compared to searches ignoring base pairing (compare columns “*RaligNAtor*” and “*RaligNAtor* (sequence only)” in Table 1). This is in particular evident when searching for families with a low degree of sequence identity. This can be explained by the small amount of information left in the RSSP for such a family, once the structural information is removed. Due to the high variability of bases in the columns of the multiple alignment of the family, the pattern contains a large number of wildcards. These symbols alone, without the constraints imposed by the base pairs, lead to unspecific patterns and therefore to a large number of false positives. We observe that, for families with sequence identity of up to 59%, the area under the curve (AUC) is considerably larger when base pairing information is taken into account. This difference decreases with increasing sequence identity (compare Figures 11(2) and (3)). Overall, the average AUC value over all families is, with a value of 0.93, still notably higher when base pairing information is considered compared to 0.89 if base pairing information is ignored (see Table 1). In this experiment, *blastn* only finds all members of those families whose sequence identity is at least 85%. This is due to the fact that *blastn* cannot appropriately handle IUPAC wildcard characters. Hence, by taking the most frequent symbol in an alignment column as consensus symbol, the heterogeneity of less conserved positions in the alignment cannot be adequately modeled. For the *blastn* searches, the average AUC value over all families is only 0.72.

### *RaligNAtor* software package

*RaligNAtor* is an open-source software package for fast approximate matching of RNA sequence-structure patterns (RSSPs). It allows the user to search target RNA or DNA sequences choosing one of the new online or further accelerated index-based algorithms presented in this work. The index of the sequence to be searched can be easily constructed with program *sufconstruct* distributed with *RaligNAtor*.

Searched RSSPs can describe any (branching, non-crossing) RNA secondary structure; see examples in Figures 1, 6, 8, and S5 of Additional file 1. Bases composing the sequence information of RSSPs can be ambiguous IUPAC characters. As part of the search parameters for RSSPs, the user can specify the cost of each sequence-structure edit operation defined above, the cost threshold of possible matches, and the number of allowed indels. The RSSPs, along with costs and thresholds per RSSP, are specified in a simple text file using a syntax that is expressive but easy to understand as shown in the mentioned figures. Another possibility is to provide the same costs and thresholds for all searched patterns as parameters in the command line call to *RaligNAtor*. To ensure maximal flexibility, the user can also define the base pairing rules from an arbitrary subset of A×A as valid pairings in a separate text file. Searches can be performed on the forward and reverse strands of the target sequence. Searching on the reverse strand is implemented by reversal of the RSSP and transformation according to Watson-Crick base pairing. Wobble pairs {(G,U), (U,G)} automatically become {(C,A), (A,C)}. Due to these transformations, the index is built for one strand only.

For describing a complex RNA with our concept of secondary structure descriptor (SSD), i.e. with multiple RSSPs, the user specifies all RSSPs in one text file. The order of the RSSPs in the file will then specify the order of the RSSP matches used to build high-scoring chains. The chain score directly depends on the score of each match occurring in the chain. This is inversely proportional to the sequence-structure edit distance of the RSSP and its matching substring in the target sequence. Hence, higher scores indicate sequences with a higher conservation which are probably more closely related to the sought RNA family.

Chaining of matches discards spurious matches not occurring in any chain. An additional filtering option eliminates matches overlapping another with a higher score for the same RSSP. This is particularly useful when indels lead to almost identical matches that are only shifted by a few positions in the target sequence.

The output of *RaligNAtor* includes not only matching positions to single RSSPs and chains, but their sequence-structure alignment to the matched substrings as well. In the *RaligNAtor* software package, all programs for searching patterns support multithreading to take advantage of computer systems with multiple CPU cores. There are two modes of parallelism. At first, different patterns are searched using multiple threads. Additionally, the search space (i.e. the sequence for the online algorithms and the index structure for the index-based methods) is partitioned, processing each part using a different thread. Lastly, we remark that our software also provides an implementation of the original algorithm of Jiang *et al.* for global sequence-structure alignment [25], easily applicable by the user.

## Conclusions

We have presented new index-based and online algorithms for fast approximate matching of RNA sequence-structure patterns. Our algorithms, all implemented in the *RaligNAtor* software, stand out from previous search tools based on motif descriptors by supporting a full set of edit operations on single bases and base pairs. See Table 2 for an overview of the algorithms. In each algorithm, the application of a new computing scheme to optimally reuse the entries of the required dynamic programming matrices and an early-stop technique to avoid the alignment computation of non-matching substrings led to considerable speedups compared to the basic scanning algorithm *ScanAlign*. Our experiments show superior performance of the index-based algorithms *LGSlinkAlign* and *LESAAlign*, which employ the suffix array data structure and achieve running time sublinear in the length of the target database. When searching for approximate matches of biologically relevant patterns on the Rfam database, *LGSlinkAlign* (*LESAAlign*) was faster than *ScanAlign* and *LScanAlign* by a factor of up to 560 (1,323) and 17 (29), respectively (see Figure 7). Comparing the two index-based algorithms, *LESAAlign* was faster than *LGSlinkAlign* when searching with tight cost threshold (i.e. sequence-structure edit distance) and no allowed indels, but became considerably slower when the number of allowed indels was increased. In this scenario, *LGSlinkAlign* was faster than *LESAAlign* by up to 4 times. In regard to the two online algorithms, *LScanAlign* was faster than *ScanAlign* by up to factor 45. In summary, *LGSlinkAlign* is the best performing algorithm when searching with diverse thresholds, whereas *LScanAlign* is a very fast and space-efficient alternative. *RaligNAtor* also allows to use the powerful concept of RNA secondary descriptors [23], i.e. searching for multiple ordered sequence-structure patterns each describing a substructure of a larger RNA molecule. For this, *RaligNAtor* integrates fast global and local chaining algorithms. We further performed experiments using *RaligNAtor* to search for members of RNA families based on information from the consensus secondary structure. In these experiments, *RaligNAtor* showed a high degree of sensitivity and specificity. Compared to searching with primary sequence only, the use of secondary structure information considerably improved the search sensitivity and specificity, in particular for families with a characteristic secondary structure but low degree of sequence conservation. We remark that, up to now, *RaligNAtor* uses a relatively simple scoring scheme. By incorporating more fine grained scoring schemes like RIBOSUM [13] or energy based scoring [42], we believe that the performance of *RaligNAtor* for RNA homology search can be further improved. Beyond the algorithmic contributions, we provide with the *RaligNAtor* software distribution, a robust, well-documented, and easy-to-use software package implementing the ideas and algorithms presented in this manuscript.

**Table 2:** Overview of the presented algorithms

**Algorithm**	**Online**	**Indexed**	**Early-stop**	**Additional memory**	**Used index tables**			
			**Acceleration**	**Requirements [bytes]**	**suf**	**lcp**	**suf**^−1^	**vtab**
*ScanAlign*	✓			0				
*LScanAlign*	✓		✓	0				
*LESAAlign*		✓	✓	5*n*	✓	✓		
*LGSlinkAlign*		✓	✓	9.125*n*	✓	✓	✓	✓

The two online algorithms *ScanAlign* and *LScanAlign* need no additional memory except for the searched sequence of length *n*. Column *additional memory requirements* refers to the additional memory needed by the used index tables. Recall that tables suf and suf^−1^ require 4*n* bytes each. Table lcp can be stored in 1*n* bytes and the bit array vtab requires only *n* bits (= 0.125*n* bytes).

## Availability

The *RaligNAtor* software package including documentation is available in binary format for different operating systems and architectures and as source code under the GNU General Public License Version 3. See http://www.zbh.uni-hamburg.de/ralignator for details.

## Competing interests

The authors declare that they have no competing interests.

## Authors’ contributions

FM and MB developed the algorithms. FM implemented the algorithms. SK implemented the chaining algorithms. MB initiated the project and provided supervision and guidance. All three authors contributed to the manuscript. All authors read and approved the final manuscript.

## Supplementary Material

Additional file 1 **Supplemental material.** Additional file 1 contains additional experiments, figures, and tables. Click here for file
