# Supplementary material for: Fast online and index-based algorithms for approximate search of RNA sequence-structure patterns
Source: BMC Bioinformatics. 2013 Jul 17;14:226. doi: 10.1186/1471-2105-14-226 (PMC3765529; doi:10.1186/1471-2105-14-226)
Supplement: Additional file 1 — Supplemental material. Additional file 1 contains additional experiments, figures, and tables. [file 1471-2105-14-226-S1.pdf]

Supplemental material for the paper: Fast online and index-based algorithms for approximate search of RNA sequence-structure patterns

Fernando Meyer Stefan Kurtz Michael Beckstette

1 Pseudocode for algorithms LESAAlign and LGSlinkAlign

The pseudocode for algorithm *LESAAlign* is given in Algorithm 1 (Figure S1). *LESAAlign* traverses the suffix array suf of the target sequence S top down, beginning with the lexicographically smallest suffix $S_{\mathsf{suf}[iSuffix]}$, where $iSuffix = 1$ at this stage. During the traversal, it computes the sequence-structure edit distance $dist(\mathcal{Q}, S_{\mathsf{suf}[iSuffix]}[1..p_{iSuffix}])$ between the RSSP $\mathcal{Q}$ and the prefix of length $p_{iSuffix}$ of each suffix $S_{\mathsf{suf}[iSuffix]}$, for $1 \leq iSuffix \leq n$. This computation is done by function *computeDP* in line 5. The input parameters of *computeDP* are the computed DP matrices, the index *iSuffix* of the current suffix, the length *iLcp* of the common prefix between the last processed suffix and the current suffix, and the last computed pattern region $\mathcal{Q}[x..y]$ denoted *lastRegion* in the code. The last two variables are used to avoid recomputation of entries of DP matrices. Function *computeDP* returns a boolean value, stored in *bMatched*, stating whether the pattern was matched, and the last newly computed region *lastRegion*. *lastRegion.r* is the right boundary of the last computed pattern region $\mathcal{Q}[x..y]$ and is used to compute variable *iLcpCheck* in line 6. *iLcpCheck*, in turn, is used to check whether suffixes of the suffix array sharing a common prefix can be skipped. If *bMatched* is true, matches are reported by function *reportMatch* in lines 8 and 18.

The pseudocode for algorithm *LGSlinkAlign* is given in Algorithm 2 (Figure S2). *LGSlinkAlign* traverses the suffix array in two combined strategies: top down and following suffix links. This is managed in the code with two main while-loops, where an outer loop (lines 3 to 47) performs the top down traversal and an inner loop (lines 13 to 46) performs the traversal via suffix links. To keep track of the last processed suffix via top down suffix array traversal, the index of this suffix is stored in variable *iSuffixTopDown*. To keep the code short, all alignment computations are performed only in the inner loop, distinguishing the strategy by which suffixes are traversed according to the boolean variable *bFollowedSuffixLink*. This variable is set to true (line 41) when the inner loop iterates and to false (line 4) when the iteration breaks. When *bFollowedSuffixLink* is false, the same *computeDP* function used by the *LESAAlign* algorithm is applied. Otherwise function *computeLastDPColumns* is applied. This function does not use lcp information, but takes advantage of the fact that the prefix of the current suffix, determined in line 36 by following a suffix link, is equal to the previously processed suffix prefix, except by its last character. This property of the suffix prefix allows to reuse already computed entries of matrices from the previously processed suffix prefix, requiring for this only one shift of the DP matrices. This is done by function *shiftDP* in line 42. While traversing the suffix array, processed suffixes are marked in the vtab table. This allows to avoid processing the same suffixes multiple times. In addition to these processed suffixes, non-contiguous suffixes of the suffix array that are known not to contain matches to RSSP $\mathcal{Q}$ are also marked in this table. This is possible when pattern $\mathcal{Q}$, for the current suffix, has an unaligned prefix of length $iUnalignedPrefixLength > 0$. For determining *iUnalignedPrefixLength* in line 31, value *lastRegion.l* is used. This value is the left boundary of the last computed pattern region $\mathcal{Q}[x..y]$. Marking the additional suffixes in vtab is performed by function *markSuffixes* (see Figure S3).

Algorithm 1: *LESAAlign*

```
input  : Index tables suf and lcp of sequence S, RSSP Q
output: Matching positions of Q in S
iSuffix := 1
iLcp := 0
lastRegion := undefined
while iSuffix ≤ n do
    (bMatched, lastRegion) := computeDP(DP, iSuffix, iLcp, lastRegion)
    iLcpCheck := lastRegion.r + d
    if bMatched then
        reportMatch(Q, S, iSuffix) //Match found at position suf[iSuffix] of S
    end
    iSuffix := iSuffix + 1
    if iSuffix ≤ n then
        iLcp := lcp[iSuffix]
    while iSuffix ≤ n and lcp[iSuffix] ≥ iLcpCheck do
        if lcp[iSuffix] < iLcp then
            iLcp := lcp[iSuffix] //Store the smallest lcp value of the skipped interval
        end
        if bMatched then
            reportMatch(Q, S, iSuffix) //Match found at position suf[iSuffix] of S
        end
        iSuffix := iSuffix + 1
    end
end
```

Figure S1: Pseudocode for algorithm *LESAAlign*. For details, see text above and the algorithm description in the main document.

This function receives as parameter a starting index *iSuffix*, *iUnalignedPrefixLength*, and the required length *iLcp* of the common prefixes of the suffixes to be marked. The function then traverses the suffix array top down and bottom up, marking all possible suffixes in vtab.

2 Influence of allowed edit costs and number of indels on search time

We describe an experiment comparing the running times of algorithms *LGSlinkAlign*, *LESAAlign*, *LScanAlign*, and *ScanAlign* to search in RFAM10.1, similar to the first benchmark described in the main document. This time we set (1) $\mathcal{K} = d$ varying the values in the interval $[0, 7]$, (2) $\mathcal{K} = 7$ varying d in the interval $[0, 7]$, and (3) $d = 0$ varying $\mathcal{K}$ in the interval $[0, 7]$. We use RSSP $\mathcal{Q}$ = (CARGAYSNVNNNNDGCRKYCCHVHRWNRUCYAG,(.(((((....((((.....))))..))))).)) of length $m = 33$ describing a stem-loop substructure of Rfam family Cripavirus internal ribosome entry site (Acc.: RF00458) [1]. The secondary structure of this family and the substructure originating the pattern can be visualized in Figure S5, where the substructure is denoted pt4. For the results of this experiment, see Figure S4 and Tables S1, S2, and S3. *LGSlinkAlign* is the fastest algorithm with measured speedup factors over *ScanAlign* (*LScanAlign*) in the range of 160.6 for $\mathcal{K} = d = 0$ to 3.3 for $\mathcal{K} = d = 7$ (17.8 for $\mathcal{K} = d = 1$ to 3.3 for $\mathcal{K} = d = 7$). In a comparison between the two online algorithms, *LScanAlign* is faster than *ScanAlign* up to a cost threshold of $\mathcal{K} = 6$ and for any value of $\mathcal{K}$ in case no indels are allowed, i.e. $d = 0$. *LESAAlign* is only faster than the online algorithms for up to $(\mathcal{K} = d) \leq 5$ and $\mathcal{K} = 7$ and $d \leq 3$. For higher cost thresholds and allowed indels, its performance decreases significantly. We explain this behavior with the increased reading depth in the suffix array implicated by $\mathcal{K}$ and d and the reduced number of suffixes sharing a common prefix that can be skipped.

Algorithm 2: *LGSlinkAlign*

```
input  : Index tables suf, lcp, suf^-1, and vtab of sequence S, RSSP Q
output: Matching positions of Q in S
iSuffixTopDown := 1
lastRegion := undefined
while iSuffixTopDown ≤ n do //Begin traversing suffix array top down
    bFollowedSuffixLink := false
    iLcp := lcp[iSuffixTopDown]
    while vtab[suf[iSuffixTopDown]] do //Skip already visited suffixes
        iSuffixTopDown := iSuffixTopDown + 1
        if iLcp > lcp[iSuffixTopDown] then //Store the smallest lcp value of the skipped interval
            iLcp := lcp[iSuffixTopDown]
        end
    end
    iSuffix := iSuffixTopDown
    while not vtab[suf[iSuffix]] do
        if bFollowedSuffixLink then //Current suffix was obtained via a suffix link
            (bMatched, lastRegion) := computeLastDPColumns(DP, iSuffix, lastRegion)
        else//Current suffix was obtained via the top-down suffix array traversal
            (bMatched, lastRegion) := computeDP(DPTopDown, iSuffix, iLcp, lastRegion)
        end
        iLcpCheck := lastRegion.r + d
        repeat
            vtab[suf[iSuffix]] := true
            if bMatched then
                reportMatch(Q, S, iSuffix) //Match found at position suf[iSuffix] of S
            end
            iSuffix := iSuffix + 1
            if iSuffix > n or vtab[suf[iSuffix]] then
                break
            end
        until lcp[iSuffix] ≥ iLcpCheck
        iSuffix := iSuffix − 1
        iUnanlignedPrefixLength := lastRegion.l − d − 1
        if iUnanlignedPrefixLength > 0 then
            markSuffixes(link(iSuffix, iUnanlignedPrefixLength), iUnanlignedPrefixLength,
            lastRegion.r + d − iUnanlignedPrefixLength)
        end
        iSuffix := link(iSuffix, 1)
        if |S_suf[iSuffix]| ≥ m − d then //If suffix is not shorter than the minimum required length
            if not bFollowedSuffixLink then
                DP := DPTopDown
            end
            bFollowedSuffixLink := true
            shiftDP(DP)
        else //Leave large while-loop and traverse suffix array top down
            break
        end
    end
end
```

Figure S2: Pseudocode for algorithm *LGSlinkAlign*. For details, see text above and the algorithm description in the main document.

Function *markSuffixes*(*iSuffix*, *iUnanlignedPrefixLength*, *iLcpCheck*)

```
//Mark suffixes by traversing suffix array top down
iSuffixDown := iSuffix + 1
while iSuffixDown ≤ n and lcp[iSuffixDown] ≥ iLcpCheck do
    if suf[iSuffixDown] − iUnalignedPrefixLength ≥ 1 then
        vtab[suf[iSuffixDown] − iUnalignedPrefixLength] := true
    end
    iSuffixDown := iSuffixDown + 1
end
//Mark suffixes by traversing suffix array bottom up
iSuffixUp := iSuffix − 1
while iSuffixUp ≥ 1 and lcp[iSuffixUp + 1] ≥ iLcpCheck do
    if suf[iSuffixUp] − iUnalignedPrefixLength ≥ 1 then
        vtab[suf[iSuffixUp] − iUnalignedPrefixLength] := true
    end
    iSuffixUp := iSuffixUp − 1
end
```

Figure S3: Function *markSuffixes* used by algorithm *LGSlinkAlign* to mark processed suffixes in table vtab. For details, see text above.

3 Comparisons between RaligNAtor and RNAMotif in terms of sensitivity and specificity

RNAMotif [2] is one of the most popular tools for approximate matching of RSSPs supporting the operations replacement and mispairing (which corresponds to the arc breaking operation defined in the main document). A number of allowed replacements and mispairings, which we here simply denote *errors*, can be specified for each part of the structure along with an overall number constraining the entire structure. However, the arc altering and arc removing operations are not supported. Also, insertions and deletions are only supported by using regular expression quantifiers. This means that the user has to know in advance for which positions of the pattern such operations can occur.

In this experiment we first analyze the results of *RaligNAtor* when searching RFAM10.1 with the tRNA (Acc.: RF00005) RSSP shown in Figure 6 of the main document. In particular, we show the importance of secondary structure information incorporated in the search for homologous sequences by varying the cost of edit operations on base pairs. Secondly, we compare the results obtained by *RaligNAtor* with the results of *RNAMotif* version 3.07 when searching with an equivalent *RNAMotif* pattern. For the used *RNAMotif* descriptor, see Figure S6.

For the searches with *RaligNAtor*, we vary the cost threshold $\mathcal{K}$ and the number of allowed indels d between 0 and 25 in steps of 5. We use operation costs $\omega_{\mathrm{d}} = \omega_{\mathrm{m}} = \omega_{\mathrm{b}} = \omega_{\mathrm{a}} = 1$ and $\omega_{\mathrm{r}} = 2$. Then we increase the costs of the operations arc breaking, arc altering, and arc removing. More precisely, we set $\omega_{\mathrm{d}} = \omega_{\mathrm{m}} = 1$, $\omega_{\mathrm{b}} = \omega_{\mathrm{a}} = 2$, and $\omega_{\mathrm{r}} = 3$. The results are shown in Table S11. Unsurprisingly, we observe that *RaligNAtor*'s sensitivity increases with increasing values of $\mathcal{K}$ and d. However, for low costs of the operations on base pairs, its specificity decreases considerably when $\mathcal{K}$ and d are increased from 20 to 25. For high costs of these operations, *RaligNAtor* is sensitive while maintaining a high specificity.

To search with *RNAMotif*, we vary the number of allowed errors per substructure between 0 and 25 in steps of 5, constraining the total number of errors to this same number. This means that no indels are allowed, since this requires many different patterns specifying possible indels only for specific pattern positions. The results are shown in Table S11. *RNAMotif* is highly specific for the complete range of allowed indels, but it is not as sensitive as *RaligNAtor*. Notably, unlike in the search with *RaligNAtor*, its sensitivity only marginally increases when the number of allowed errors varies from 20 to 25, with some decrease of its specificity. Similar results can be obtained with *RaligNAtor* by setting $d = 0$.

$\mathcal{K} = d$	#matches	*ScanAlign*	*LScanAlign*	*LESAAlign*	*LGSlinkAlign*
0	4	261.80	14.60	0.56	1.63
1	23	270.43	43.83	3.62	2.46
2	71	282.99	67.87	12.39	6.06
3	164	291.66	124.34	43.19	19.90
4	354	300.93	203.04	125.74	43.01
5	3,771	323.66	256.16	246.60	66.25
6	86,509	326.85	294.44	348.69	83.96
7	1,546,439	339.55	342.66	459.26	104.08

Table S1: Times in minutes required by algorithms *ScanAlign*, *LScanAlign*, *LESAAlign*, and *LGSlinkAlign* to search with a stem-loop pattern of length 33 in RFAM10.1. Times are influenced by the cost threshold $\mathcal{K}$ and the number of allowed indels d. For a graphical representation of the measurements, see Figure S4(1).

$\mathcal{K}$	d	#matches	*ScanAlign*	*LScanAlign*	*LESAAlign*	*LGSlinkAlign*
7	0	398	264.68	211.03	101.11	37.93
7	1	2,873	275.95	240.55	159.24	45.92
7	2	23,440	281.80	262.52	216.26	56.46
7	3	103,792	290.80	278.29	268.33	68.04
7	4	309,464	302.53	295.05	317.23	78.01
7	5	688,675	325.10	313.40	364.49	86.72
7	6	1,434,360	333.96	325.80	409.53	95.51
7	7	1,546,439	339.55	342.66	459.26	104.08

Table S2: Times in minutes required by algorithms *ScanAlign*, *LScanAlign*, *LESAAlign*, and *LGSlinkAlign* to search with a stem-loop pattern of length 33 in RFAM10.1. Here, the cost threshold $\mathcal{K}$ is constant and the number of allowed indels d increases progressively.

4 RNA family classification using Structator

Structator [3] is an ultra fast tool for RSSP matching. It is the first tool to integrate algorithms for global and local chaining of RNA pattern matches. However, it has limited support to approximate matching, lacking support of the sequence-structure edit operations allowed by *RaligNAtor*.

Here, we report the number of sequence members obtained by *Structator* when searching RFAM10.1 with the SSDs of families RF00458 and RF01736. The SSDs are shown in Figures S5 and 8 of the main document. Despite sharing the same pattern syntax with *RaligNAtor*, we observe the following differences and adaptations.

- *Structator* cannot search for stem-loop patterns with dangling ends. Therefore, we remove the dangling end of the RSSP ires3 belonging to the SSD of family RF00458.
- As *Structator* does not allow for edit operations, parameters *cost* and *indels* have no effect in the search. However, a number of allowed mispairings for each pattern can be specified by the user. We allow for each pattern a number of mispairings equal to the value of parameter *cost*.
- *Structator* has lower sensitivity compared to *RaligNAtor* when the latter searches with allowed costs greater than zero. For this reason, we chain the matches to the single RSSPs varying the minimum required chain length between 2 and the total number of RSSPs of each SSD.

The results are shown in Table S10. We observe that, in the search with the SSD of family RF00458, *Structator* cannot find all its true sequence members without increasing considerably the number of false positives. In the search with the SSD of family RF01736, only up to 4 true sequence members can be found. *RaligNAtor*, in contrast, finds all sequence members of both families and no false positives, as described in the main document.

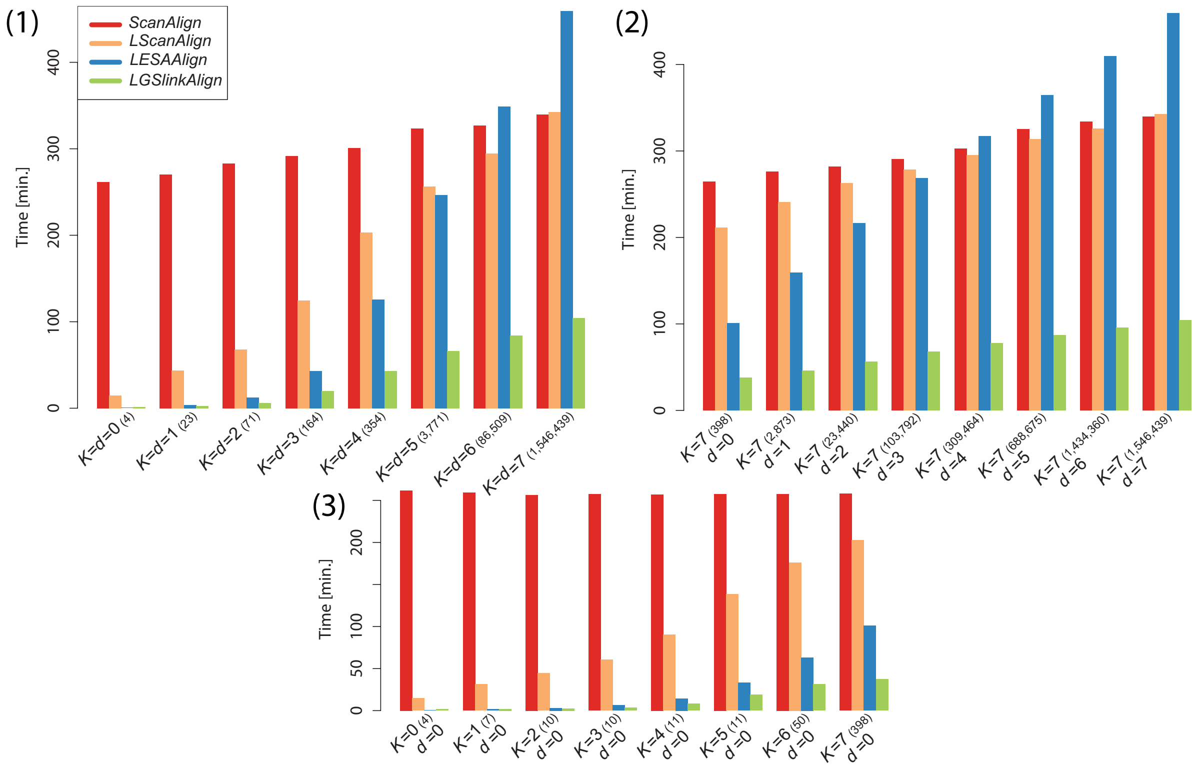

Figure S4: Running times needed by the different algorithms to search with a stem-loop pattern of length 33 in RFAM10.1. In (1) the cost threshold $\mathcal{K}$ and the number of allowed indels d increase equally. In (2) $\mathcal{K} = 7$ is constant and d increases from 0 to 7. In (3) $d = 0$ is constant and $\mathcal{K}$ increases from 0 to 7. The numbers of resulting matches are given on the x-axes in brackets.

$\mathcal{K}$	d	#matches	*ScanAlign*	*LScanAlign*	*LESAAlign*	*LGSlinkAlign*
0	0	4	261.80	14.60	0.56	1.63
1	0	7	259.17	31.45	1.78	1.86
2	0	10	256.27	44.37	3.10	2.52
3	0	10	257.19	60.41	6.43	3.57
4	0	11	257.01	90.22	14.52	8.07
5	0	11	257.61	138.30	33.11	18.90
6	0	50	257.50	176.02	63.11	31.45
7	0	398	258.00	202.68	100.82	37.45

Table S3: Times in minutes required by algorithms *ScanAlign*, *LScanAlign*, *LESAAlign*, and *LGSlinkAlign* to search with a stem-loop pattern of length 33 in RFAM10.1. Here, indels are not allowed and the cost threshold $\mathcal{K}$ increases progressively.

$\mathcal{K} = d$	#matches	*ScanAlign*	*LScanAlign*	*LESAAlign*	*LGSlinkAlign*
0	1	1,582.03	21.81	0.53	3.09
1	168	1,581.86	50.36	2.53	3.81
2	900	1,643.86	68.26	5.95	13.17
3	3,050	1,670.71	100.22	16.22	30.29
4	9,274	1,710.75	141.12	42.23	43.66
5	28,603	1,759.80	196.09	90.61	64.74
6	77,805	1,830.33	319.32	198.94	107.63

Table S4: Times in minutes required by algorithms *ScanAlign*, *LScanAlign*, *LESAAlign*, and *LGSlinkAlign* to match in RFAM10.1 the single RSSP describing the consensus secondary structure of the tRNA (Acc.: RF00005). Times are influenced by the cost threshold $\mathcal{K}$ and the number of allowed indels d.

$\mathcal{K}$	d	#matches	*ScanAlign*	*LScanAlign*	*LESAAlign*	*LGSlinkAlign*
6	0	10,516	1,536.08	123.18	17.69	22.82
6	1	30,633	1,576.73	156.50	35.67	39.87
6	2	49,287	1,657.61	188.79	58.99	52.98
6	3	64,226	1,703.31	222.36	86.39	65.94
6	4	74,146	1,754.08	256.78	119.47	80.55
6	5	77,679	1,808.84	287.49	156.48	94.03
6	6	77,805	1,830.33	319.32	198.94	107.63

Table S5: Search times in minutes required by algorithms *ScanAlign*, *LScanAlign*, *LESAAlign*, and *LGSlinkAlign* to match in RFAM10.1 the single RSSP describing the consensus secondary structure of the tRNA (Acc.: RF00005). Here, the cost threshold $\mathcal{K}$ is constant and the number of allowed indels d increases progressively.

$\mathcal{K}$	d	#matches	*ScanAlign*	*LScanAlign*	*LESAAlign*	*LGSlinkAlign*
0	0	1	1,582.03	21.81	0.53	3.09
1	0	166	1,601.82	35.39	1.21	2.86
2	0	439	1,601.20	45.20	2.05	3.00
3	0	1,112	1,601.90	54.83	2.87	3.72
4	0	2,963	1,606.61	74.29	4.90	5.71
5	0	6,518	1,601.01	96.93	9.53	11.57
6	0	10,516	1,601.93	118.26	17.34	21.87

Table S6: Search times in minutes required by algorithms *ScanAlign*, *LScanAlign*, *LESAAlign*, and *LGSlinkAlign* to match in RFAM10.1 the single RSSP describing the consensus secondary structure of the tRNA (Acc.: RF00005). Here, no indels are allowed and the cost threshold $\mathcal{K}$ increases progressively.

RFAM10.1 subset	*ScanAlign*			*LScanAlign*			*LESAAlign*			*LGSlinkAlign*		
size (MB)	flg1	flg2	flg3	flg1	flg2	flg3	flg1	flg2	flg3	flg1	flg2	flg3
98.3	37.42	40.32	20.61	17.42	16.78	6.27	18.17	11.90	2.20	5.86	4.91	0.91
196.7	74.91	81.51	41.21	34.55	33.18	12.29	29.53	19.45	3.46	9.69	8.15	1.44
295.0	111.63	120.53	60.29	51.54	50.20	18.35	38.70	25.09	4.33	13.05	11.01	1.89
393.4	146.50	155.24	78.69	68.93	67.10	24.92	45.46	30.13	5.21	16.07	13.57	2.31
491.7	179.22	191.24	97.46	87.00	83.32	30.68	52.46	34.78	6.10	19.01	16.05	2.97
590.1	213.99	230.11	117.29	103.18	99.96	37.06	58.87	39.32	6.84	21.59	18.08	3.29
688.4	251.42	269.40	138.52	121.99	117.08	43.04	65.68	43.48	7.50	24.26	20.64	3.74
786.8	287.32	310.06	157.28	137.78	134.40	48.83	71.18	47.52	8.27	26.47	22.56	4.05

Table S7: Search times in minutes used to investigate the scaling behavior of algorithms *ScanAlign*, *LScanAlign*, *LESAAlign*, and *LGSlinkAlign* on random subsets of RFAM10.1 of increasing size. See the definition of the searched RSSPs flg1, flg2, and flg3 and further details of this experiment on the main document.

RSSP	*ScanAlign*	*LScanAlign*	*LESAAlign*	*LGSlinkAlign*
ires1	13.13	12.85	1.02	2.68
ires2	203.67	356.78	135.03	60.12
ires3	51.21	8.54	0.37	1.61
ires4	281.44	103.52	28.11	14.53
ires5	138.86	103.90	22.35	13.31

Table S8: Times in minutes required by algorithms *ScanAlign*, *LScanAlign*, *LESAAlign*, and *LGSlinkAlign* to match the RSSPs that build the SSD for family Cripavirus internal ribosome entry site (Acc.: RF00458) in RFAM10.1.

RSSP	*ScanAlign*	*LScanAlign*	*LESAAlign*	*LGSlinkAlign*
flg1	288.21	143.23	74.90	27.03
flg2	310.68	141.73	50.01	22.00
flg3	156.60	51.74	8.67	3.83

Table S9: Times in minutes required by algorithms *ScanAlign*, *LScanAlign*, *LESAAlign*, and *LGSlinkAlign* to match the RSSPs that build the SSD for family flg-Rhizobiales RNA motif (Acc.: RF01736) in RFAM10.1.

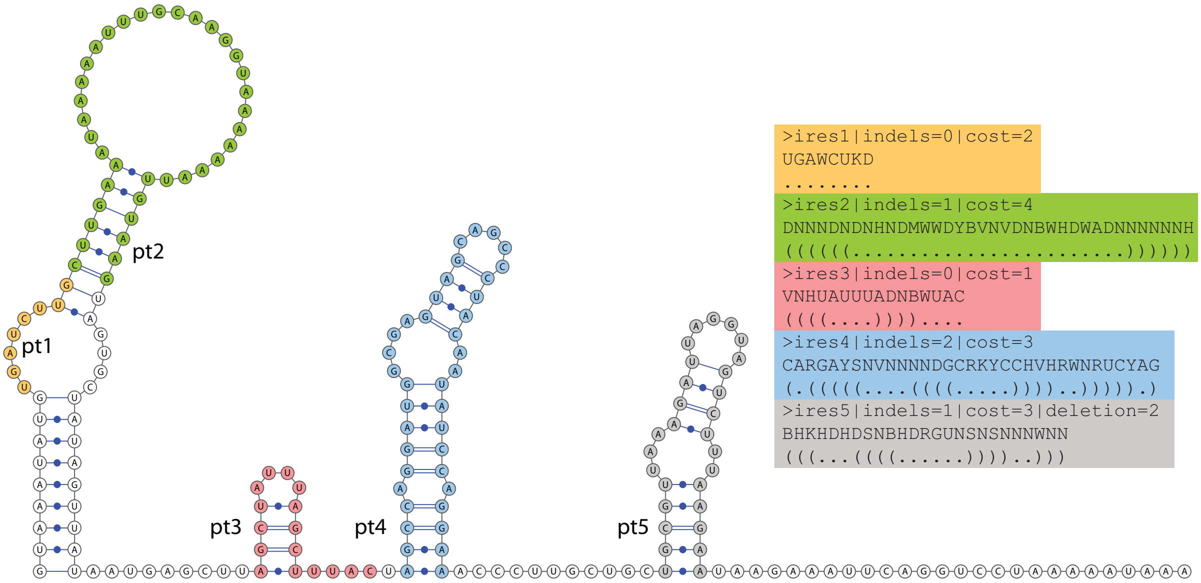

Figure S5: Consensus secondary structure of family Cripavirus internal ribosome entry site (Acc.: RF00458) showing its four characteristic stem-loop substructures pt2, pt3, pt4, and pt5 and the moderately conserved strand pt1 as drawn by *VARNA* [4]. The secondary structure descriptor (SSD) for this family, on the right-hand side, consists of five RSSPs ires1, ires2, ires3, ires4, and ires5 describing the strand and stem-loop substructures.

RF00458			
Min. chain length	#TP	#FP	#FN
2	16	5807	0
3	16	14	0
4	14	0	2
5	3	0	13

RF01736			
Min. chain length	#TP	#FP	#FN
2	4	0	11
3	1	0	14

Table S10: Results obtained with *Structator* [3] when searching with the secondary descriptors of families RF00458 and RF01736 in RFAM10.1. The first column for each family indicates the minimum required length of a chain to be considered a matching chain. #TP, #FP, and #FN stand for number of true positives, false positives, and false negatives, respectively. For additional details, see text above.

```
parms
    wc += gu;

descr
    h5(seq="^GSSVVYR$")
      ss(seq="^UR$")
      h5(seq="^GYYY$")
        ss(seq="^ARYUGGUUA$")
      h3(seq="^RMRC$")
      ss(seq="^R$")
      h5(seq="^YYDSV$")
        ss(seq="^YUBHHAM$")
      h3(seq="^BCHRD$")
      ss(seq="^WRRUY$")
      h5(seq="^RYRGG$")
        ss(seq="^UUCRAWU$")
      h3(seq="^CCYDY$")
    h3(seq="^HNBBNSY$")
    ss(seq="^R$")
```

Figure S6: *RNAMotif* descriptor without errors for the tRNA.

RaligNAtor, edit operation costs: $\omega_{\rm d} = \omega_{\rm m} = \omega_{\rm b} = \omega_{\rm a} = 1$, $\omega_{\rm r} = 2$

$\mathcal{K} = d$	#TP	#FP	#FN	Sensitivity	Specificity	Accuracy	Precision
0	1	0	1,101,832	0.000	1.000	0.600	1.000
5	10,726	0	1,091,107	0.010	1.000	0.606	1.000
10	146,124	3	955,709	0.133	1.000	0.671	1.000
15	517,984	65	583,849	0.470	1.000	0.822	1.000
20	959,243	164,708	142,590	0.871	0.941	0.921	0.853
25	1,097,783	1,168,140	4,050	0.996	0.702	0.767	0.484

RaligNAtor, edit operation costs: $\omega_{\rm d} = \omega_{\rm m} = 1$, $\omega_{\rm b} = \omega_{\rm a} = 2$, $\omega_{\rm r} = 3$

$\mathcal{K} = d$	#TP	#FP	#FN	Sensitivity	Specificity	Accuracy	Precision
0	1	0	1,101,832	0.000	1.000	0.600	1.000
5	10,427	0	1,091,406	0.009	1.000	0.606	1.000
10	127,865	2	973,968	0.116	1.000	0.662	1.000
15	263,277	8	838,556	0.239	1.000	0.722	1.000
20	669,252	262	432,581	0.607	1.000	0.874	1.000
25	1,034,028	122,285	67,805	0.938	0.956	0.951	0.894

RNAMotif

#Errors	#TP	#FP	#FN	Sensitivity	Specificity	Accuracy	Precision
0	1	0	1,101,832	0.000	1.000	0.600	1.000
5	7,289	0	1,094,544	0.007	1.000	0.604	1.000
10	40,669	0	1,061,164	0.037	1.000	0.621	1.000
15	66,451	1	1,035,382	0.060	1.000	0.633	1.000
20	68,236	1	1,033,597	0.062	1.000	0.634	1.000
25	68,492	139	1,033,341	0.062	1.000	0.634	0.998

Table S11: Results of the searches in RFAM10.1 for the tRNA (Acc.: RF00005). For the two series of searches with *RaligNAtor* using the operation costs above, the sequence-structure pattern shown in Figure 6 of the main document is used. For the searches with *RNAMotif* varying the number of allowed errors (#Errors), the descriptor shown in Figure S6 is used. These errors comprehend replacements and mispairings. #TP, #FP, and #FN stand for number of true positives, false positives, and false negatives, respectively. Sensitivity is computed as $\frac{\#TP}{\#TP+\#FN}$, specificity as $\frac{\#TN}{\#TN+\#FP}$, accuracy as $\frac{\#TP+\#TN}{\#TP+\#FP+\#FN+\#TN}$, and precision as $\frac{\#TP}{\#TP+\#FP}$. For additional details, see text above.

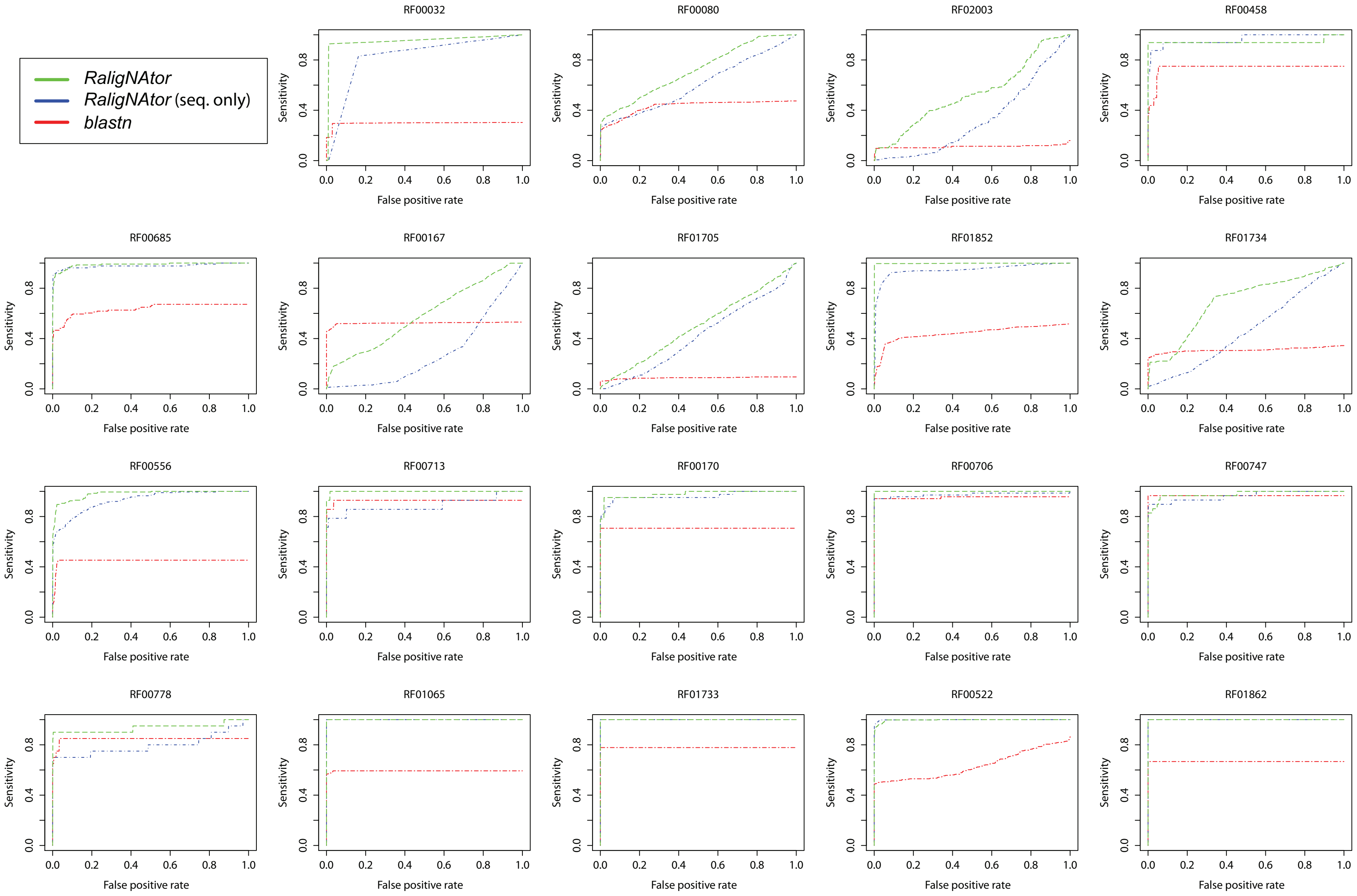

Figure S7: Results of ROC analyses using *RaligNAtor* with and without base pairing information and *blastn* [5] for the Rfam families shown in Table 1 of the main document. ROC curves showing *RaligNAtor*'s classification performance using (ignoring) base pairing information are shown in green (blue). Blast performance results are shown in red. The ROC curves are sorted by increasing level of sequence identity of the respective family, i.e. in the same order each family is listed in Table 1 of the main document. Additional ROC curves are shown in Figure S8. For details of this experiment, see corresponding description in the main document.

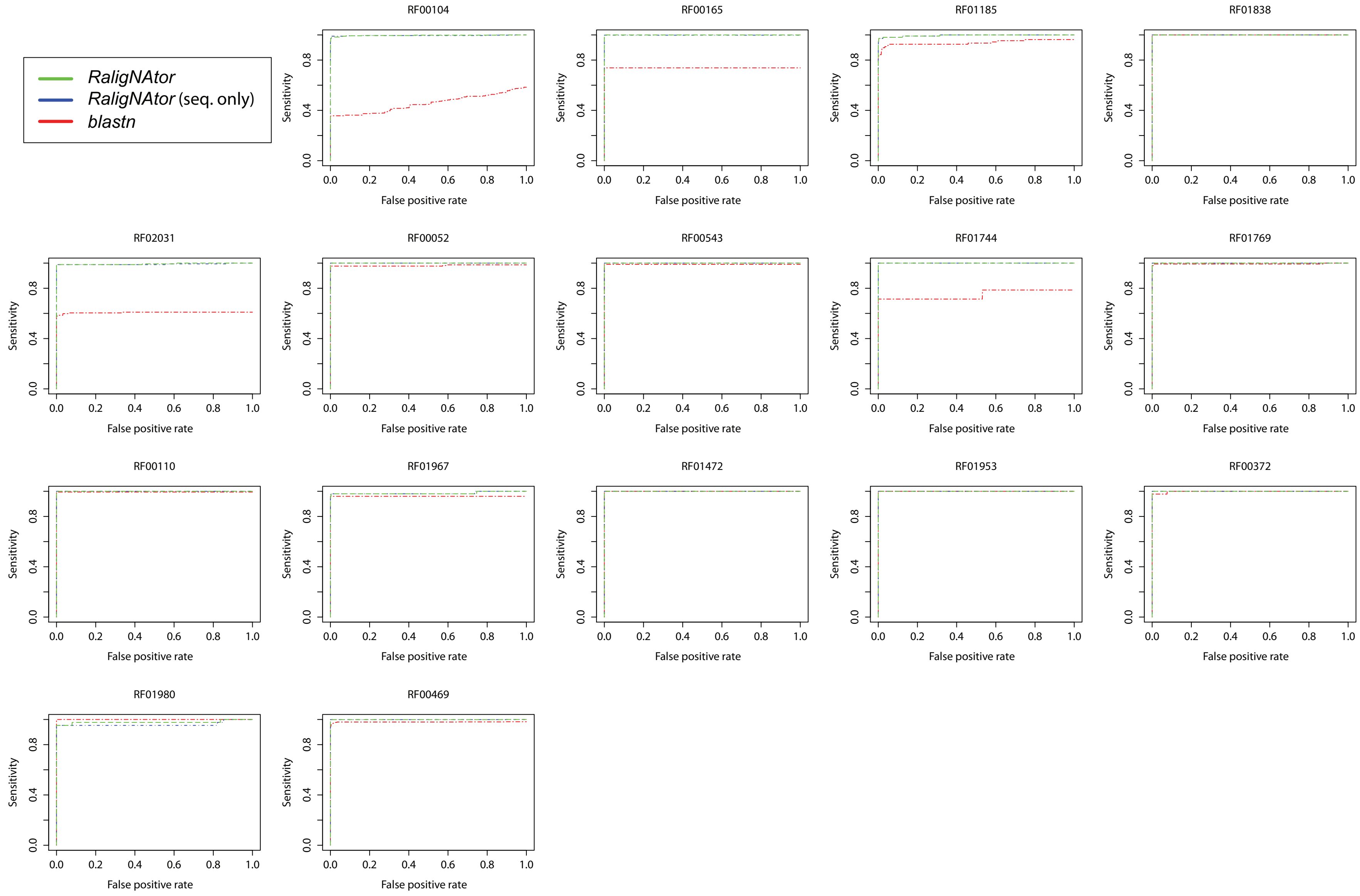

Figure S8: Additional ROC curves. See description of Figure S7 for details.

References

[1] Y. Kanamori and N. Nakashima. A tertiary structure model of the internal ribosome entry site (IRES) for methionine-independent initiation of translation. *RNA*, 7(2):266–274, 2001.

[2] T. Macke, D. Ecker, R. Gutell, D. Gautheret, D.A. Case, and R. Sampath. RNAMotif – A new RNA secondary structure definition and discovery algorithm. *Nucleic Acids Res.*, 29(22):4724–4735, 2001.

[3] F. Meyer, S. Kurtz, R. Backofen, S. Will, and M. Beckstette. Structator: fast index-based search for RNA sequence-structure patterns. *BMC Bioinformatics*, 12(1):214, 2011.

[4] K. Darty, A. Denise, and Y. Ponty. VARNA: Interactive drawing and editing of the RNA secondary structure. *Bioinformatics*, 25(15):1974–1975, 2009.

[5] S. F. Altschul, T. L. Madden, A. A. Schäffer, J. Zhang, Z. Zhang, W. Miller, and D. J. Lipman. Gapped BLAST and PSI-BLAST: a new generation of protein database search programs. *Nucleic Acids Res.*, 25(17):3389–3402, 1997.
